# Thermography for disease detection in livestock: A scoping review

**DOI:** 10.3389/fvets.2022.965622

**Published:** 2022-08-09

**Authors:** Rosemary McManus, Lisa A. Boden, William Weir, Lorenzo Viora, Robert Barker, Yunhyong Kim, Pauline McBride, Shufan Yang

**Affiliations:** ^1^Division of Pathology, Public Health and Disease Investigation, School of Veterinary Medicine, College of Medical Veterinary and Life Sciences, University of Glasgow, Glasgow, United Kingdom; ^2^Global Academy of Agriculture and Food Systems, The Royal (Dick) School of Veterinary Studies, The Roslin Institute, University of Edinburgh, Edinburgh, United Kingdom; ^3^Scottish Centre for Production Animal Health and Food Safety, School of Veterinary Medicine, College of Medical Veterinary and Life Sciences, University of Glasgow, Glasgow, United Kingdom; ^4^School of Physical Sciences, University of Kent, Canterbury, United Kingdom; ^5^Information Studies Department, School of Humanities, University of Glasgow, Glasgow, United Kingdom; ^6^School of Law, University of Glasgow, Glasgow, United Kingdom; ^7^School of Computing, Edinburgh Napier University, Edinburgh, United Kingdom

**Keywords:** surveillance, veterinary, disease, thermography, livestock, infra-red

## Abstract

Infra-red thermography (IRT) offers potential opportunities as a tool for disease detection in livestock. Despite considerable research in this area, there are no common standards or protocols for managing IRT parameters in animal disease detection research. In this review, we investigate parameters that are essential to the progression of this tool and make recommendations for their use based on the literature found and the veterinary thermography guidelines from the American Academy of Thermology. We analyzed a defined set of 109 articles concerned with the use of IRT in livestock related to disease and from these articles, parameters for accurate IRT were identified and sorted into the fields of camera-, animal- or environment-related categories to assess the practices of each article in reporting parameters. This review demonstrates the inconsistencies in practice across peer-reviewed articles and reveals that some important parameters are completely unreported while others are incorrectly captured and/or under-represented in the literature. Further to this, our review highlights the lack of measured emissivity values for live animals in multiple species. We present guidelines for the standards of parameters that should be used and reported in future experiments and discuss potential opportunities and challenges associated with using IRT for disease detection in livestock.

## Introduction

### Early detection of animal disease

Early disease detection is critical to avert health and socio-economic impacts for livestock and agricultural sectors. Animal diseases can be categorized into ‘production,' ‘endemic,' and ‘exotic' classes, important concepts in terms of animal welfare, health and economics. This review explores thermal imaging as a methodology to improve early warning systems, to facilitate better identification and/or discrimination of diseased vs. non-diseased animals, timely collection of data and rapid analysis and communication. Early detection is a crucial factor in animal and zoonotic disease preparedness and control. Swift and decisive action in response to disease incursion and spread is necessary to protect animal and human health, farmer livelihoods and the wider economy. Mandatory infectious disease reporting to national authorities and endemic disease surveillance play critical roles in the prevention and control of disease. When an infectious disease outbreak occurs in livestock, there can be significant negative impacts on trade and the economy in the affected country. For example, the 2001 foot and mouth disease (FMD) outbreak in the UK cost an estimated £3.1 billion to the agriculture and food sectors and the UK Bovine Spongiform Encephalopathy (BSE) crisis is estimated to have cost the country £3.7 billion (1). Wider social impacts have also been documented, such as loss of public confidence in animal products (2), adverse mental health in agricultural workers (3) and food insecurity (4).

### Infra-red thermography

There has been a great deal of research into infra-red thermography (IRT) as a potential tool to aid the early detection of important animal diseases. Infra-red Thermography uses infrared (IR) imaging to collect temperature-related data. Infra-red sensors detect radiation in the long-IR range of the electromagnetic spectrum and produce images of that radiation, called thermograms. The amount of radiation emitted by an object increases with temperature, allowing variations in temperature to be detected. An elevated core body temperature and/or an increase in temperature regionally on an animal are key indicators for many infectious diseases and inflammatory processes. Core temperatures are usually taken *per rectum*, but this method is time-costly and labor-intensive, especially in extensively managed herds. If temperature measurement were carried out remotely and in an automated manner, this would likely (a) reduce the time to detect disease, (b) reduce overall farmer costs and (c) improve overall animal husbandry and welfare by enabling more rapid treatment. Reliable, non-invasive, automated, remote sensing systems utilizing IR technology would enable easier monitoring of early indicators of animal health aberrations. Such systems would have clear utility in veterinary bio-surveillance and biosecurity programs but would need to be reliable, accurate, cost-effective and user-friendly to be implemented and attract user uptake. Repeated calls to augment early warning systems for disease surveillance with real-time applications that can be integrated into animal health policy and disease control pathways have been made (5, 6). Hence, accurate IRT used as a non-invasive temperature measurement tool for livestock has the potential to be a cost- and time-saving technique that would propel both the animal health sector and the precision livestock industry into the future.

### Accuracy, methods and reporting standards

Harrap et al. (7) published a systematic review of thermographic methods employed in biological research. The review described the ideal requirements for accurate thermographic measurements: (i) a well-focused image of the target; (ii) an emissivity measurement of the object being imaged; (iii) a reflected temperature measurement of the target; (iv) an environmental temperature measurement; (v) a relative humidity measurement and (vi) a distance measurement between the camera and the target, noting that omission of parameters can drastically impair accuracy. Emissivity is a measure of the efficiency of a surface to emit thermal energy relative to a perfect ‘blackbody' source. It represents the ratio of the energy radiated from a material's surface to that radiated from a perfect emitter (a blackbody) at the same temperature and wavelength and under the same viewing conditions. A blackbody is a body or surface that absorbs all radiant energy at all wavelengths and has an emissivity value of 1. Incomplete information being presented on thermographic parameters was attributed to one of two scenarios: that the IR camera was used correctly with appropriate parameter adjustment, but that details were omitted in the published methodology, or, that the camera was used incorrectly and consequently, parameters were not adjusted or reported (7). Many technical aspects are critical in the field of IRT. Partial errors result from uncertainties in the parameters used to estimate the real temperature: emissivity, reflected temperature, transmittance, ambient temperature, camera response and calibrator (blackbody) temperature accuracy. It is therefore imperative that, whenever possible, these parameters are controlled. Emissivity, ambient and reflected temperature, and relative humidity are usually determined by the user and manually input into the thermal camera to be included in internal IR calculations. Typically, ±2°C or ±2% uncertainty of the reading is quoted as the accuracy of thermal cameras. This refers to how close the camera is to providing the true temperature value. The estimation is obtained using the ‘Root-Sum-of-Squares' uncertainty analysis technique. The total measurement error (Δ*T*) is estimated from partial errors for each parameter involved (Δ*T*_*i*_), following the equation below:


$$\begin{array}{l} \Delta T=\sqrt{}\Delta T_{1}^{2}+\Delta T_{2}^{2}+\ldots +\Delta T_{n}^{2} \end{array}$$

This prescribes how total measurement error is calculated based on individual parameter errors. Understanding the impact each of the individual parameters has on the uncertainty is, therefore, crucial in establishing the reliability of temperature measurement and thus the true efficacy of IRT. To date, no paper has highlighted all possible parameters which should be considered in attempts to reduce partial errors. This study aims to address this knowledge gap with a view to improving accuracy of IRT for livestock disease detection.

### Existing guidelines

The American Academy of Thermology (AAT)^1^ created guidelines to aid the performance of veterinary thermography. Several areas where IRT is appropriate are outlined with the following being most applicable to disease detection: (i) as a diagnostic aid where changes in the thermal patterns of scanned regions of interest suggest a regional or systemic diagnosis whereby anatomic imaging modalities can then be used to characterize the nature of the problem; and (ii) as a method to enhance the clinical assessment of the veterinary patient. Boundaries within which IRT should be undertaken are offered and these have been considered in the discussion of individual parameters in this section. The guidelines state that IRT is highly sensitive to environmental conditions and therefore an understanding of these is imperative. IRT is contraindicated if certain environmental parameters, such as sunlight, ambient temperature and the presence of draft, cannot be controlled. Further contraindications for use include the absence of relevant expertise (infrared radiation physics), the inability to evaluate bilaterally symmetrical images and the involvement of an uncooperative patient. These guidelines represent the current basis for standardization of thermographic methods in animals.

### Aims of review

To investigate essential IRT parameters for disease detection in livestock, we undertook a review of the relevant IRT literature to assimilate the full range of parameters used in infrared thermography. Based on this approach, we: (i) investigate knowledge gaps in the field, with a focus on livestock emissivity values; (ii) critically examine the parameters necessary to optimize IRT for disease detection in livestock and make recommendations for minimum operating and reporting standards of experimental parameters; and (iii) discuss the current direction of experimental research in this field and predict future trends.

## Methods

This section outlines the search criteria, the eligibility criteria, the review process, and the parameters extracted from each study included in the review.

### Search criteria

A literature search was carried out using *Web of Science* (Clarivate Analytics), covering papers published between 1864 and 2021 using all databases comprising the Web of Science Core Collection, CABI: CAB Abstracts, BIOSIS Citation Index, BIOSIS Previews, Current Contents Connect, MEDLINE, Derwent Innovations Index, Russian Science Citation Index, Zoological Record, SciELO Citation Index, Data Citation Index, KCI-Korean Journal Database. The final search was undertaken on the 22nd September 2021. The search of a wide range of dates and databases was undertaken to include any foundational articles concerning IRT methods and their use in agricultural settings.

The following search query was employed, using English as the search language: [TS = (cow^*^ OR cattle OR calf OR calves OR bovine OR bovid^*^ OR horse OR equid^*^ OR ruminant^*^ OR goat^*^ OR sheep OR pig^*^ OR deer OR pony OR ponies) AND TS = (fever OR disease OR mastitis OR lame^*^) AND TS = (“infrared thermography” OR “infra-red thermography” OR irt OR “thermal imaging”)], where “TS” denotes “Topic” and “^*^” denotes all derivations of a word. Multiple words concerning livestock were included to ensure the search captured all derivations or words in common use to describe cattle, horses, sheep, goats and pigs. Additional literature was also sourced *via* bibliographies of articles found in the initial *Web of Science* search and unstructured searches of other academic databases, namely Google Scholar and Scopus.

### Eligibility criteria

Articles were deemed eligible for inclusion in the review if they were a primary research article or communication, published in a peer-reviewed journal, reporting thermographic studies in livestock and containing relevant imagery or data. Inclusion criteria are outlined in Table 1.

**Table 1:** Inclusion criteria.

	**Inclusion criteria**	**Notes**
**Topic**	Infra-red thermography studies with data collected as images or video	Infra-red thermometry studies were excluded. These are studies which utilize an IR thermometer to digitally display a temperature, but do not display a visual thermogram
**Sector**	Livestock, e.g., agricultural animals or food-producing animals	Companion animals, wild animals and birds were excluded
**Purpose**	Disease detection in livestock	Studies concerned with other physiological processes such as stress, heat, fear and reproduction were excluded
**Type**	Peer-reviewed studies published in journals. Conference publications that are not isolated abstracts where a comprehensive methods section is provided	Isolated abstracts from conferences not containing a methods section were excluded
**Rigor**	Peer-reviewed studies not retracted by the publishing body at the time of the last search	Publications retracted by the publishing body at the time of the last search were excluded
**Language**	Published in English	

### Review process

Search results were examined in chronological order and publications were cross-referenced against inclusion criteria (Table 1). This process meant that only research papers that reported work utilizing IRT for disease detection in livestock were retained in the study. Data on IRT methodology and use, and reporting of relevant parameters were extracted manually, described and assessed against the inclusion criteria. After completion of the full review process, search results were examined for a second time to ensure accuracy and consistency.

### Parameters and definitions

Parameters were classified according to the following categories: ‘camera,' ‘animal,' and ‘environmental' parameters. Descriptions of these parameters are included in Tables 2–**4**.

**Table 2:** Camera parameters.

**Datapoint**	**Description**
Camera make and model	The manufacturer and model of the thermal camera(s) used in the publication
Spectral range	The range of wavelengths that the thermal camera can measure in μm
Calibration	Whether calibration of the thermal camera was performed or not
Calibration method	The method of calibration provided in the manuscript; denoted as ‘n/a' if this does not apply
Temperature range	The span of temperatures between the minimum and maximum temperatures that the camera can measure in degrees Celsius (°C)
Video or image	Whether video or static image data was collected
Video frame rate	The video frame rate of the thermal camera; denoted as ‘n/a' if the paper used still imagery
Resolution	The image resolution of the thermal camera in pixels per inch
Field-of-view	The maximum area of a sample that a camera can image
Instantaneous field-of-view	The measure of the spatial resolution of a remote sensing imaging system; defined as the angle subtended by a single detector element on the axis of the optical system
Lens	Details of the lens of the thermal camera
Thermal sensitivity (noise equivalent temperature difference)	A measure of how well a thermal imaging detector can distinguish between very small differences in thermal radiation in the image, expressed in millikelvin (mK)
Focus	Details of the focus of the thermal camera
Focal length	The focal length of the lens
Spot size	The area each pixel covers on the target
Drift	Thermal inaccuracies caused by camera components
Stability	Thermal inaccuracies over time
Accuracy	The supplied accuracy of the thermal camera
Fixed/handheld	An indication of whether the camera was fixed in position or was handheld by an operator

#### Camera parameters

Parameters and definitions pertaining to the thermal camera used in each publication were extracted and are outlined in Table 2. Missing parameters were classified as ‘not recorded' if they were not reported in the paper.

#### Environmental parameters

Parameters and definitions pertaining to the environment in each publication were extracted from each paper and are outlined in Table 3. Missing parameters were classified as ‘not recorded' if they were not reported in the paper.

**Table 3:** Environmental parameters.

**Datapoint**	**Description**
Ambient temperature considered	An indication of whether ambient temperature was considered
Ambient temperature recorded	The ambient temperature that was recorded
Relative humidity considered	An indication of whether relative humidity was considered
Relative humidity recorded	The relative humidity that was recorded
Reflected temperature considered	An indication of whether reflected temperature was considered
Sheltered from direct sunlight	An indication of whether the research was carried out in a sheltered environment or in direct sunlight
Angle considered	An indication of whether angle to the area of interest was considered
Angle recorded	The angle to the area of interest that was recorded in the manuscript
Distance	An indication of whether distance from the area of interest was considered
Distance recorded	The distance from the area of interest that was recorded
Wind considered	An indication of whether wind speed was considered
Rain considered	An indication of whether presence of rain was considered

#### Animal-related parameters

Parameters and definitions pertaining to the animals used in each publication were extracted from each paper and are outlined in Table 4. Missing parameters were classified as ‘not recorded' if they were not reported in the paper.

**Table 4:** Animal-related parameters.

**Datapoint**	**Description**
Species	The species of the animal(s) of interest
Emissivity (ε)	The ε value used
Emissivity (ε) value source	The source cited for the ε value (i.e., independently measured or citation of another publication)
Region of interest	The body region of interest of the animal for thermal imaging
Stress	An indication of whether the manuscript noted any stress effects on the research animals
Coat or skin color	An indication of whether the manuscript noted the coat or skin color of the research animals; denoted as 'not recorded' if this was not provided
Coat length	An indication of whether the manuscript noted the length of the coat/hair of the research animals; denoted as 'not recorded' if this was not provided
Age	The age of the animal(s) of interest

## Results

### Literature search

**Figure 1:**
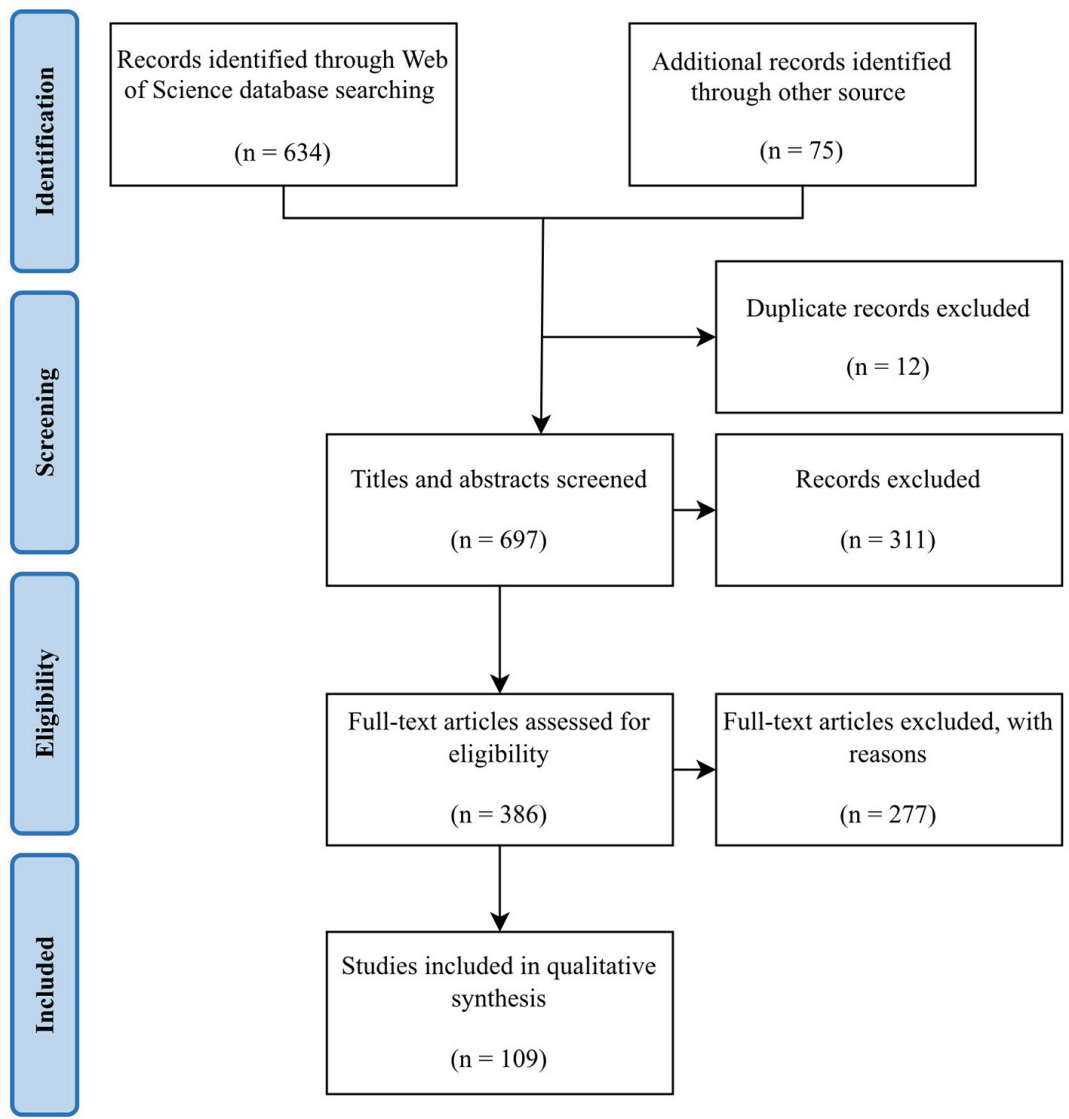
Search approach detailing the number of records identified, included and excluded.

The literature search identified published articles that utilized IRT in the livestock sector and extracted the methodology for thermography in each manuscript to assimilate the range of parameters used. The *Web of Science* search yielded a total of 634 results and extended literature searching yielded another 75 articles. After 12 duplicates were removed, a total of 697 records remained. Further screening of titles and abstracts resulted in the exclusion of 311. The full texts of the remaining 386 articles were assessed and a further 277 articles were excluded. As a result, 109 articles were included in the final review (Figure 1).

### Parameter reporting and opportunities to minimize partial errors

This section outlines the variation in parameter reporting over time together with a breakdown of the animal, camera and environmental parameters reported. These results highlighted the lack of measured values for emissivity in livestock.

#### Variation in parameter reporting over time

**Figure 2:**
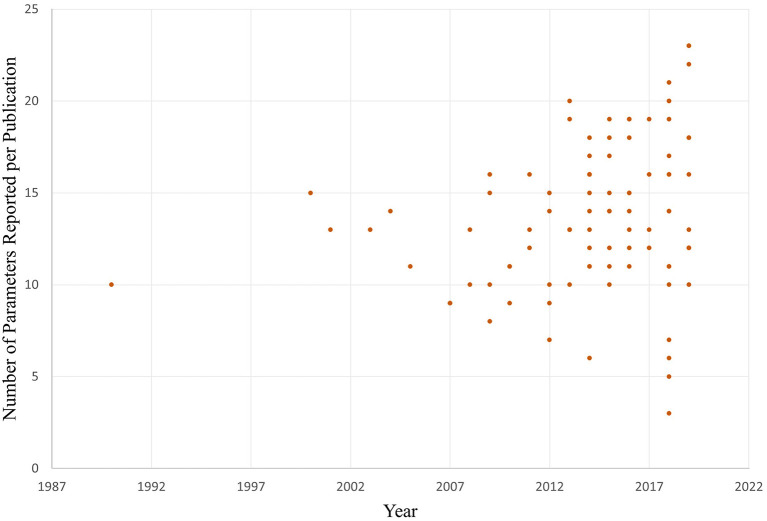
Number of parameters reported per publication over time.

The number of parameters reported by each article included in the review is shown in Figure 2 between 1990 to 2021. Parameter reporting ranged from 3 to 23 out of a possible maximum of 39 with a mean of 14, a median of 14 and a mode of 13.

#### Animal-related parameters

**Figure 3:**
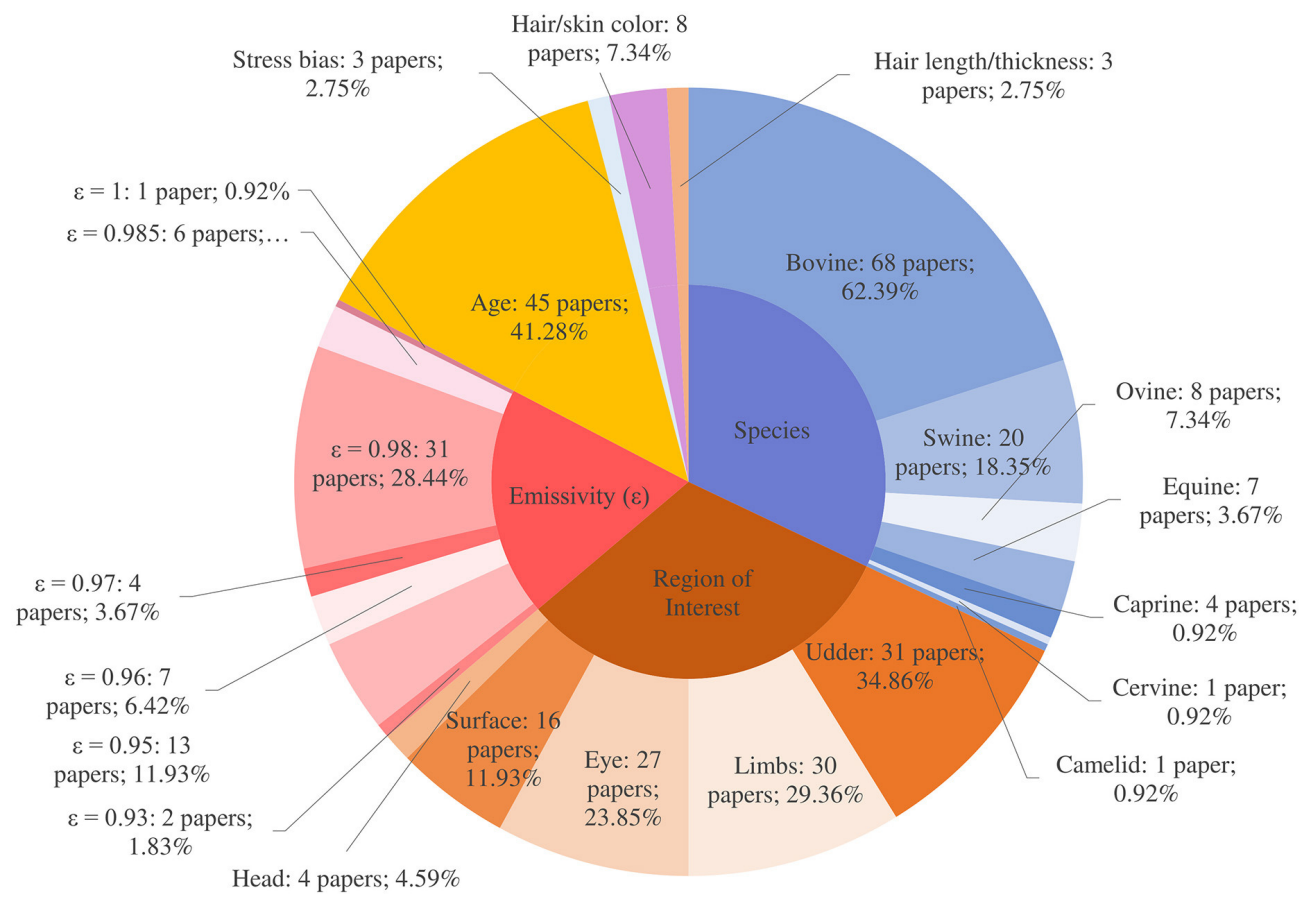
Animal-related parameter reporting.

Animal-related parameters included: emissivity, emissivity value source, coat/skin color and length, stress, species and the region of interest (ROI) on the animal. Across the review publications, this corresponded to 872 instances where parameters could have been reported (8 parameters × 109 papers). However, there were only 406 instances of parameters being reported (46.56%). Animal-related parameters were associated with more recorded ‘datapoints' across the review compared to environmental and camera categories (Figure 3).

##### Emissivity

**Figure 4:**
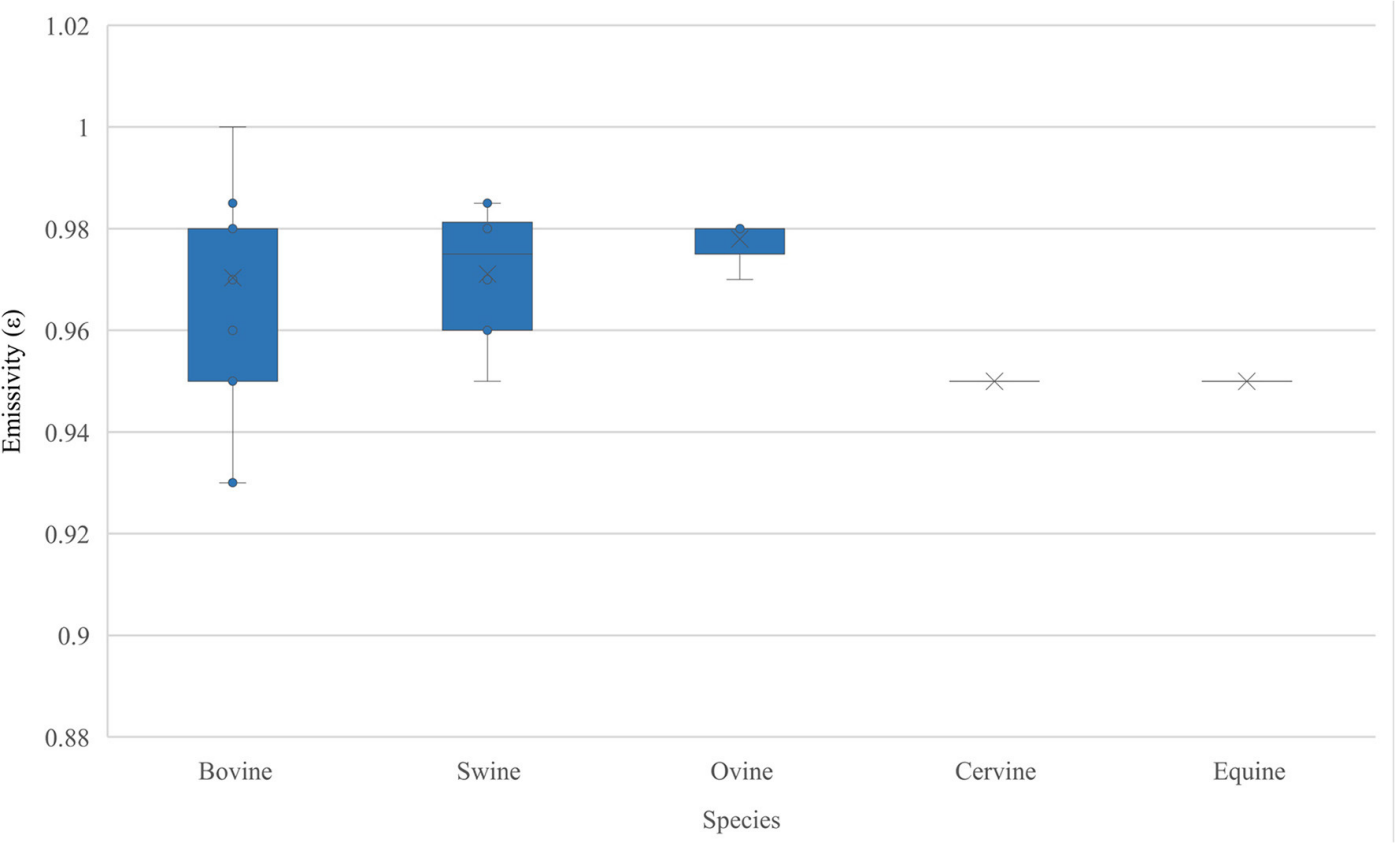
Range of emissivities (ε) used across species in the articles in the review.

Emissivity values were reported in 64 studies (58.72%) (8–71) with 45 studies (72–116) not stating the emissivity value used. Figure 4 shows the range of emissivity values used for each species in the review, ranging from 0.93 to 1.0. Of the 64 studies that stated emissivity, just 21 provided a source for the value used (10, 11, 14, 15, 20, 22, 30, 33, 36, 37, 42, 43, 47, 50, 51, 54, 55, 58, 66, 68, 69). Sources for these emissivity values were not immediately clear from the publication and this is explained below. These 21 studies cited 24 references (14, 32, 50, 54, 61, 66, 117–134) with some studies citing more than one reference and some references being cited multiple times. Three studies (37, 50, 68) reference the IR camera user manual (offering the emissivity for living tissues of between 0.95 and 0.98)^2^^,^ ^3^^,^ ^4^, and three of the references were books (128, 130, 132). Some of these source studies were included in the 109 publications identified in this review (13, 14, 32, 50, 54, 66, 123, 127). However, most of these papers were found to fall outside the scope of the review as they were not concerned with the application of IRT to livestock disease.

**Figure 5:**
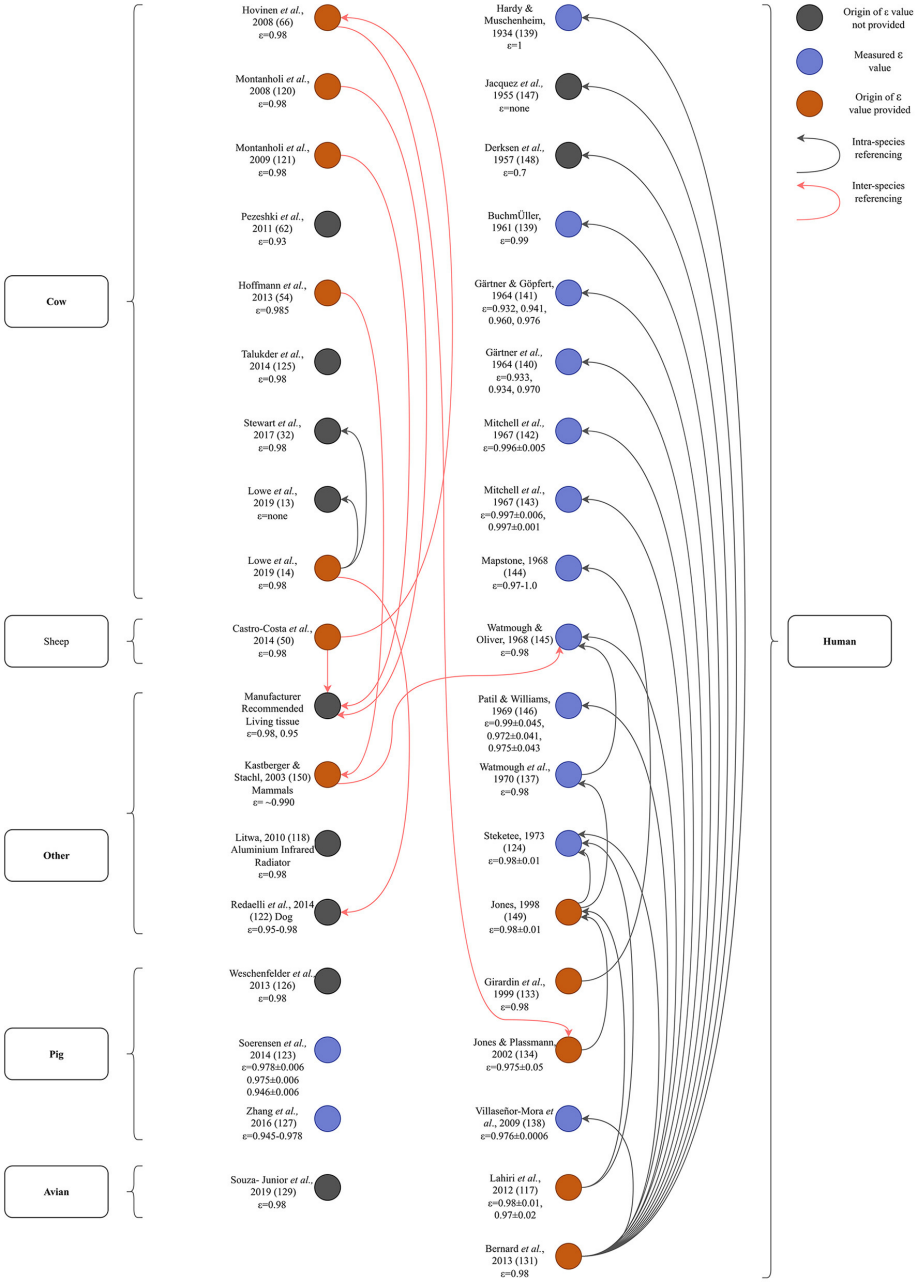
Diagram illustrating the origins of emissivity values found in the studies included in the review. Out of 109 papers included in this review, 21 papers provided 27 sources for their emissivity value (20 original research articles, one review article, three books and three references to the camera's manufacturer's manual). These referenced sources and the sources which informed them are mapped out here to ascertain the ε values stated, what species they referred to and whether the values used could be traced to a measured ε value. Arrow direction indicates referencing of older articles. The first author's name, the year of publication and the emissivity value used (if present) are included. For papers that could not be classified based on ‘species,' the object is stated. For ease of illustration, only the original research articles and the manufacturer's manual that were referenced are included in the diagram. This figure illustrates that most primary sources are human emissivity values and only two values have been measured in livestock, both in pigs.

The range of emissivity values (0.93–1.0) used within each species illustrates a lack of consensus (Figure 4). Measured values for emissivity in bovine, ovine, cervine and equine species are absent (Figure 5). Soerensen et al. (123) and Zhang et al. (127) evaluated skin emissivity of pigs, obtaining similar results (123, 127). Soerensen et al. found that ear base, udder, and shoulder (with hair) were 0.978±0.006, 0.975±0.006 and 0.946±0.006, respectively, and also discovered that skin with blood perfusion had higher emissivity than without and that clipping the hairs of the shoulder tended to increase emissivity (123). Room temperature was found to have an insignificant effect on the calculated emissivity value (123). Zhang et al. found that surface emissivity varied from 0.945 to 0.978, with the highest value (0.978) being obtained from the ear base and shoulder (127). Experiments used to determine emissivity in both studies were based on data collected from sedated and dead animals, and this is likely to have impacted the results due to the influence of sedation on peripheral blood perfusion.

##### Knowledge gap: Emissivity

Camera emissivity settings play a key role in IR temperature estimation. Emissivity is a measure of the efficiency of a surface to emit thermal energy relative to a perfect ‘blackbody' source. Emissivity is the ratio of the energy radiated from a material's surface to that radiated from a perfect emitter (a blackbody) at the same temperature and wavelength and under the same viewing conditions. A blackbody is a body that absorbs all radiant energy at all wavelengths and has an emissivity of 1. Conversely, a body which reflects all radiant energy at all wavelengths has a value of 0. Emissivity is highly dependent on the target object's surface morphology, roughness, oxidation, spectral wavelength, temperature, and the angle from which it is viewed. Teledyne FLIR's (the largest thermal imaging systems company in the world) user manual^5^ suggests an emissivity measurement method, whereby IR images of the target are taken with and without black insulating electrical tape stuck to the target (which has a known emissivity of 0.97). Emissivity is set to 0.97 when the tape is attached and to 1 when it is not. Further images are then taken without the tape, adjusting the emissivity setting on the camera to match the temperature obtained without the tape to the one obtained with the tape. However, this method is only valid when the surface temperature exceeds the ambient temperature by at least 10°C as may be anticipated in temperate regions. In tropical climes, where animal surface temperatures and ambient temperatures may be within 10°C of each other, this method is unlikely to yield accurate results. While this method is still in current use in human testing (135), the ability to test this method with livestock is dependent on the temperament of the animal and whether it allows this procedure to be carried out.

A network diagram was constructed to elucidate the origins of the emissivity values used in the studies included in this review (Figure 5). From this, it can be observed that the majority of independently measured values for emissivity are for human (*n* = 12) (124, 136–146) rather than livestock tissues with the exception of two measurements in pigs (123, 127). A number of sources (*n* = 13, including manufacturer user's manuals)^6^^,^ ^7^ offer no independently measured or referenced source for the used emissivity value stated (13, 32, 62, 118, 122, 125, 126, 129, 147, 148) and a number of sources (*n* = 12) did not independently measure emissivity but referenced another source for the value they used (14, 50, 54, 66, 117, 120, 121, 131, 133, 134, 149, 150).

Nearly all emissivity research (Figure 5) can be traced back to two primary papers (124, 145). Both studies independently measured emissivity, but in human tissues. Watmough and Oliver used an IR camera with a spectral range of 2.0 to 5.4 μm, concluding that the emissivity of human skin was greater than 0.98 (145). However, the researchers did not account for ambient temperature and had they made this correction, they would have concluded that skin emissivity lies between 0.94 and 1.0, within 2.0 to 5.4 μm (151). Steketee measured skin emissivity using a monochrometer (an optical instrument which measures the light spectrum) and a thermometer (124). A reference blackbody source formed part of the monochrometer which could be maintained at the same temperature as that of the skin. Skin temperature was measured using a compensated method in which a fine gold ring with a thermocouple was warmed so that its temperature did not change when it touched the skin. The effect of ambient radiation was substituted by adjusting the radiation from another reference source in the monochrometer with which incident radiation was switched in an alternating fashion by chopper blades. The average observed emissivity was 0.98±0.01, within 3 to 14 μm. The experiments were well-designed, the principle being a comparison of the radiation from the skin and that from a blackbody at the same temperature. Because both quantities are close to each other, a small difference in each measurement would cause a considerable error in the difference of the two quantities (151). Togawa stated that previously reported skin emissivity values were unlikely to be as reliable as results obtained by a method based on reflectance measurement upon transient stepwise change in the ambient radiation temperature (151). Togawa's study concluded that within the range of 8 to 14 μm, the emissivities of normal and intact skin of humans from multiple body sites would be about 0.97 on average, and that differences in emissivity for different regions of interest of the body are insignificant (151). This was improved by Huang and Togawa by applying “least-squares fitting” to 64 IR images instead of only two thermograms as in the previous study but was not appropriate for use in living animals (152). Sanchez-Marin et al. refined this method based on the calculation of the difference of two IR images, with one image acquired prior to the projection of a CO_2_ laser beam on the surface of the skin and another after the projection, with the difference between the images being used as the reflectance (153). From this, they were able to estimate emissivity (153).

But is emissivity really that important? In a study evaluating the effect of multiple factors on IR eye temperature in cattle, Church et al. (98) experimented with changing the emissivity setting on the camera and found that adjusting the value by 0.05 altered the resulting IR temperature by 0.5°C. In a disease surveillance system where the aim is to discriminate between ‘positive' (hyperthermic) and ‘negative' (normothermic) animals, a difference of 0.1°C may signify the presence or absence of disease. This is the magnitude of difference appreciated by veterinary practitioners when using a rectal thermometer. Emissivity ranged from 0.93 to 1.0 across the studies included in the review, yielding a difference of 0.08 between the top and bottom of the range. As much of the research being conducted is concerned with absolute temperatures and using cut-off points to define a disease, it is critical to use accurate emissivity. Polat et al. did not report an emissivity value, despite IRT cut-off points for diagnosis of sub-clinical mastitis being defined (104). This lack of an emissivity value demonstrates an insufficient understanding of critical parameters when utilizing IRT in the context of disease surveillance. Table 5 illustrates the effect a change in emissivity can have on resulting temperatures using the Stefan-Boltzman constant. From Table 5, it can be observed that a change of 0.01 to emissivity (ε) can lead to a systematic bias of 0.69 to 0.76°C. Dos Santos Sousa et al. defined a cut-off point of 30.0°C to detect laminitis using IRT of the hoof (64). This cut-off temperature yielded a sensitivity of 96%, a specificity of 63% and an epidemiological accuracy of 75% when compared with the gold standard of using hoof testers for diagnosis. Emissivity was set at 0.98. As a working guide, in the experiments carried out by dos Santos Sousa et al. (64), we find that ε = 0.98±0.01 will imply T = 30.0±0.74°C. Within the range of ε determined in this review (0.93 to 1.0), analyses that employ a hard cut-off temperature point will also be systematically biased, if error of emissivity is not taken into account.

**Table 5:** The errors in resulting IR temperature if emissivity is altered for a given temperature of 30.0°C to demonstrate that change of emissivity (ε) = 0.01 can lead to a systematic bias of 0.69 to 0.76°C where *E* = energy flux (also called the radiant emittance), ε = emissivity, σ = Stefan–Boltzmann constant (5.67 × 10–8 W/ m^2^ K^4^).

**Emissivity (ε)**	***E* (W/m^2^)**	***δT* coefficient**	***δT* (°C), *δε* = 0.01**	**δT (°C), *δε* = −0.01**	***δT* (°C), *δε* = 0.05**
1	478.9	−75.8	−0.76	0.76	−3.79
0.99	474.1	−74.8	−0.75	0.75	−3.74
0.98	469.3	−73.9	−0.74	0.74	−3.69
0.97	464.5	−73.0	−0.73	0.73	−3.65
0.96	459.7	−72.0	−0.72	0.72	−3.60
0.95	454.9	−71.1	−0.71	0.71	−3.55
0.94	450.1	−70.1	−0.70	0.70	−3.51
0.93	445.3	−69.2	−0.69	0.69	−3.46

See Supplementary Data for supporting calculations.

What emissivity value is most appropriate for livestock? The AAT recommends using a value of 0.98 (human skin) unless a different emissivity is both known and accepted for the animal and ROI under study. McGowan et al. (154) reported that most mammal emissivities used thus far in research are derived from Hammel (155). Hammel measured the emissivity of the pelage (hair-covered tissue, pelts cut from dead mammals) of ten species of dead arctic fauna, finding that all furs had emissivity values between 0.98 and 1.00. Hammel used a method described by Hardy (136) where a contact probe was used to determine skin temperature, and in order to reduce the effect of ambient air on the contact probe, the room temperature was raised until it approximated that of the skin. This method has since been proven inadequate for emissivity measurement (151). McGowan et al. set out to find values for mammals, focusing on pelage and obtained the following values for livestock: Thoroughbred horse: 0.90±0.03, Goat: 0.89±0.02, Friesian cow: 0.79±0.03 and Charolais cow: 0.88±0.03, which are much lower than any values currently in use in IR research in livestock (154). Mean pelage emissivity of sampled specimens was 0.86±0.01 and there was neither a relationship between emissivity and taxonomy, nor between emissivity and hair metrics (154). The authors concluded that single (0.98) or narrow ranges (0.95 to 1.00) of emissivity values are likely inappropriate for obtaining accurate mammal IR surface temperature readings (154). This contradicted the findings of Soerensen et al. (123) and Zhang et al. (127) who carried out emissivity experiments in pigs using different methods. Soerensen et al. (123) used needle probes attached to a data logger to estimate skin temperature of multiple skin sites with emissivity set to 1 on the IR camera. Images were analyzed after collection to estimate emissivities. However, the same study found that skin with blood perfusion had a higher emissivity than without and that removal of hair from the area to be examined increased emissivity, so it remains to be determined which is the most appropriate value for emissivity as these experiments were carried out on non-living tissue with hair (123). Zhang et al. (127) built on earlier human emissivity research (152, 156) and estimated surface emissivity in real-time in living animals by changing the surface reflection energy of animals and a reference body and thus altering the ambient radiant energy without confirming the actual temperature of the animal surface. This was a non-invasive approach, unlike Soerensen et al.'s work, so may have more utility in future research. It is also possible that there are differences in emissivity between species, individuals of the same species and regions of interest on the same animal. There is therefore a need to establish usable values for livestock animals.

##### Skin/hair color and hair thickness

Although few studies recorded coat or hair color (*n* = 8; 7.34%) or thickness of hair (*n* = 3; 2.75%), both are important drivers of surface temperature. Darker cows naturally absorb more heat from the sun whereas white cows reflect more heat radiation. Dark coated cows thus have a higher surface temperature on a warm day. The type and color of coat changes the skin's ability to radiate and absorb heat. A thicker coat will also lead to greater levels of insulation. Anzures-Olvera et al. (157) evaluated the impact of coat color on temperature in cattle, finding that the rectal temperature of black cows was 0.1°C higher than white cows, but that coat color did not alter body surface temperature readings measured by IRT. In outdoor field trials, the effect of direct sunlight was strongly dependent on hair color, having a profound effect on IRT temperature with white areas of cow's heads producing temperatures of ~35°C, while black areas produced temperatures of ~50°C (98). Dirt also affects surface temperature measurements, affecting the ability of the surface to radiate energy and to conduct heat. Metzner et al. (52) found that surface temperature was markedly lower in the presence of dirt. Udder surface temperatures are affected by the amount of hair present (115), this deviation being most pronounced in German Simmental cattle which have a lot of udder hair (115). Corresponding temperatures were lower than non-haired udders as a result (115). Soerensen et al. found that clipping the hairs of the shoulder tended to increase emissivity in pigs (123) and Giro et al. (11) discussed hair length with regard to cattle breed and used a breed with a lower hair density in their study to eliminate the interference of hair with heat elimination from the body and consequent IRT temperature.

##### Region of interest

Although region of interest (ROI) was reported in nearly all studies (*n* = 108; 99.08%), there is no single ROI which emerges as the most important or useful for health monitoring and disease detection in livestock. The most popular ROIs to be studied included the udder (*n* = 31; 34.86%) and limbs (*n* = 30; 29.36%) together with the eye (*n* = 27; 23.85%), body surface (*n* = 16; 11.93%) and head (*n* = 4; 4.59%), with many studies investigating multiple ROIs. Studies performing IRT on the udder, limbs and eye were mainly concerned with cattle (*n* = 25; 22.94%, *n* = 21; 19.27% and *n* = 20; 18.35%, respectively). In contrast to this, IRT was more commonly used for monitoring body surface temperatures in the context of studies relating to pigs (*n* = 10; 9.17%), the remainder being concerned with cattle (*n* = 5; 4.59%) and sheep (*n* = 1; 0.92%). Of these papers, 75% were concerned with young animals, namely piglets, calves and lambs, where IRT was applied to thermoregulation capabilities in neonates (55, 96), vaccine response (89) and diseases specific to young animals (13, 27).

A recent paper documented body surface temperature patterns for Jersey dairy cattle in a thermoneutral environment (158). IRT images of the left and right periocular area, right and left eye, caudal left foreleg, cranial left foreleg, right and left flank and forehead were sampled. Of these regions, forehead temperature was shown to have the greatest correlation with rectal temperature (*r*^2^ = 0.35). In another study aimed at determining the most reliable body area for measurement of rectal temperature in pregnant and non-pregnant cows, the strongest correlation was found using the thorax (*r*^2^ = 0.377) with the udder and the eyeball showing slightly weaker correlations (*r*^2^ = 0.357 and *r*^2^ = 0.316 respectively) (77). Thermogram temperature was also shown to differ between seasons and reproductive phases. Stumpf et al. documented a strong correlation between lateral udder and rectal temperatures in cattle (*r*^2^ = 0.897) (68), demonstrating that the udder area has great promise as an indicator of core body temperature. Respiratory rate in calves has also been investigated using IRT; the rate of flank movements as captured by optical imaging was compared with IRT of thermal fluctuations around nostrils (14) and, encouragingly, these readings were found to be highly correlated. Given the prevalence and economic importance of pneumonia in young cattle, a role could be envisaged for using IRT for respiratory disease surveillance in this class of stock.

Multiple investigations into using eye measurements as a proxy for rectal temperature have been carried out in animals with induced respiratory and diarrheal disease (100, 110, 112) with more recent work examining correlations between rectal temperature and IRT readings from (i) the eye (maximal value; *r*^2^ = 0.37); (ii) the muzzle (*r*^2^ = 0.28); and (iii) the medial canthus of the eye (*r*^2^ = 0.27) (9). Numerous options for the ‘eye' ROI have been previously adopted, thus Shu et al. sought to standardize the ROI for measuring eye temperature. Mean and maximum temperatures of five orbital ROIs (medial canthus, lateral canthus, eyeball, whole eye and lacrimal sac) were manually captured and the maximum temperature obtained at the lacrimal sac of the left eye was found to provide the highest correlation coefficients with respiration rate (*r*^2^ = 0.6) and rectal temperature (*r*^2^ = 0.52). Future research should focus on using maximal readings from this specific locus rather than the entire ocular area.

##### Stress

Stress is one of the main parameters that can influence an animal's temperature, but it was not well-reported in the literature (*n* = 3; 2.75%). The hypothalamic-pituitary-axis (HPA) can lead to stress-induced hyperthermia when stimulated by the sympathetic system when an animal is stressed. In an induced stress study in cows, Stewart et al. (159) measured eye temperature and tested serum cortisol to ascertain if increased cortisol (indicating activation of the HPA related to stress) correlated with increased eye temperature. It was found that exogenous stimulation of the HPA axis did not increase eye temperature although an increase following catheterization was detected, suggesting there is a cognitive component to stress. It could be hypothesized that an animal may only need to perceive the situation as stressful for eye temperature to increase, meaning habituation to a certain procedure/object could potentially reduce this effect. Gomez et al. (160) investigated whether IRT of visible eye white and eye temperature measurements were feasible physiological indicators of acute stress in cows. Maximum eye temperature did not differ between stress and control treatments, but the authors found that the IR values differed between breeds. In Brown Swiss cows, maximum eye temperature increased during and after both treatments, whereas in Red Holstein cows, it increased after (but not during) both treatments, which was attributed to differences in eye coloration patterns (160). Stress affects the animal's temperature and so needs to be considered when clinically interpreting the temperature. While this contrasts with other animal-related parameters which account for inaccuracies in the measured temperatures, stress must be considered in advance of future experiments.

#### Camera parameters

**Figure 6:**
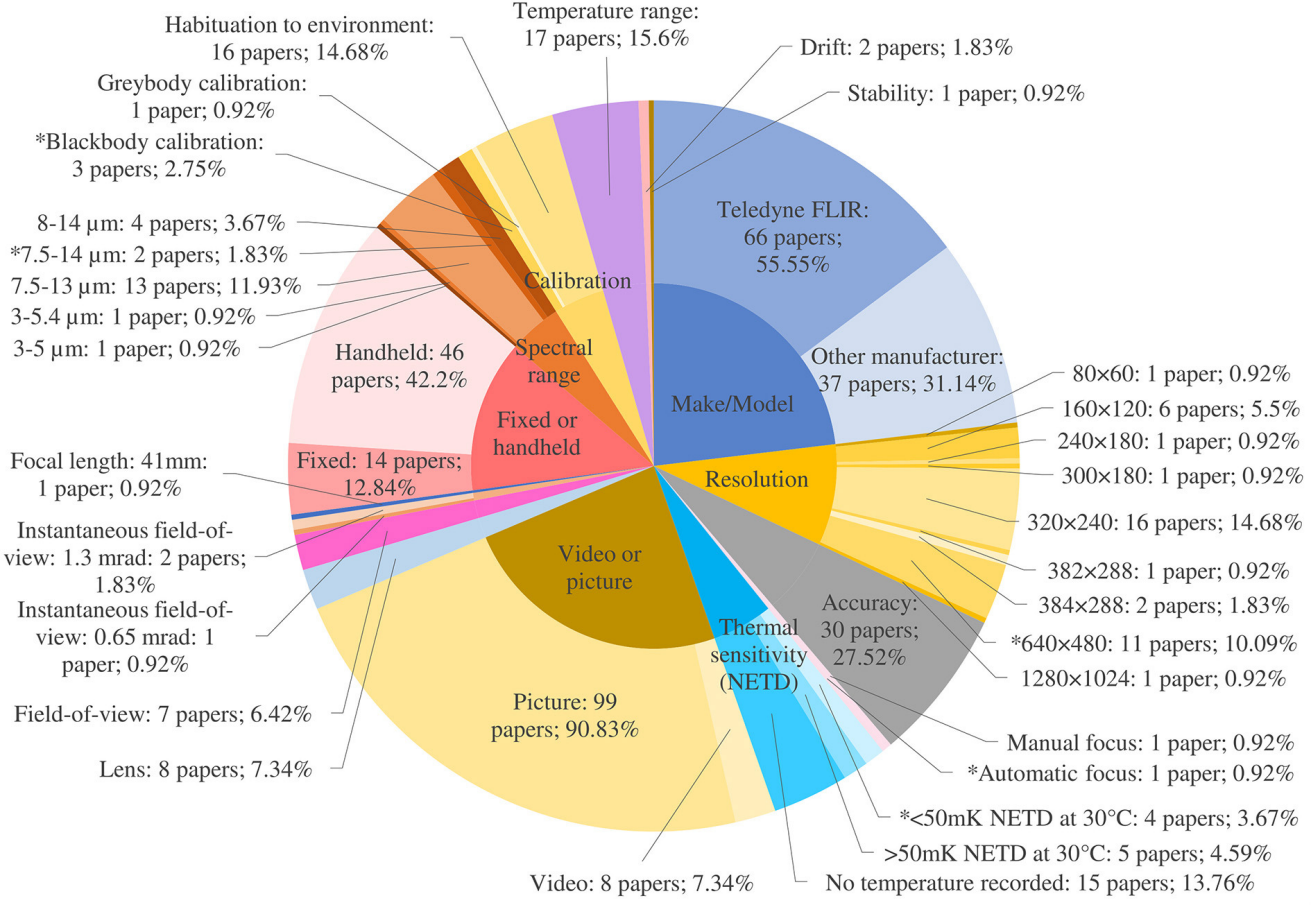
Camera parameter reporting where ^*^ denotes the recommended value. Recommended values for instantaneous field-of-view and spot size are ≤2.6 mRad and 2.1 ×2.0 mm (3 × 3 or 9 pixels) at 40 cm distance from the animal respectively.

Camera specifications including spectral and temperature range, field-of-view, instantaneous field-of-view, resolution, thermal sensitivity and focus are important when choosing a suitable device for IRT of animals. We collected information regarding reporting of 19 camera parameters. This corresponded to 2071 instances where parameters could have been reported (19 parameters × 109 papers). However, only 470 instances of parameters being reported (22.69%) were recorded (Figure 6). Despite the importance of camera parameters, they were reported less frequently than animal-related and environmental parameters.

##### Spectral range

**Figure 7:**
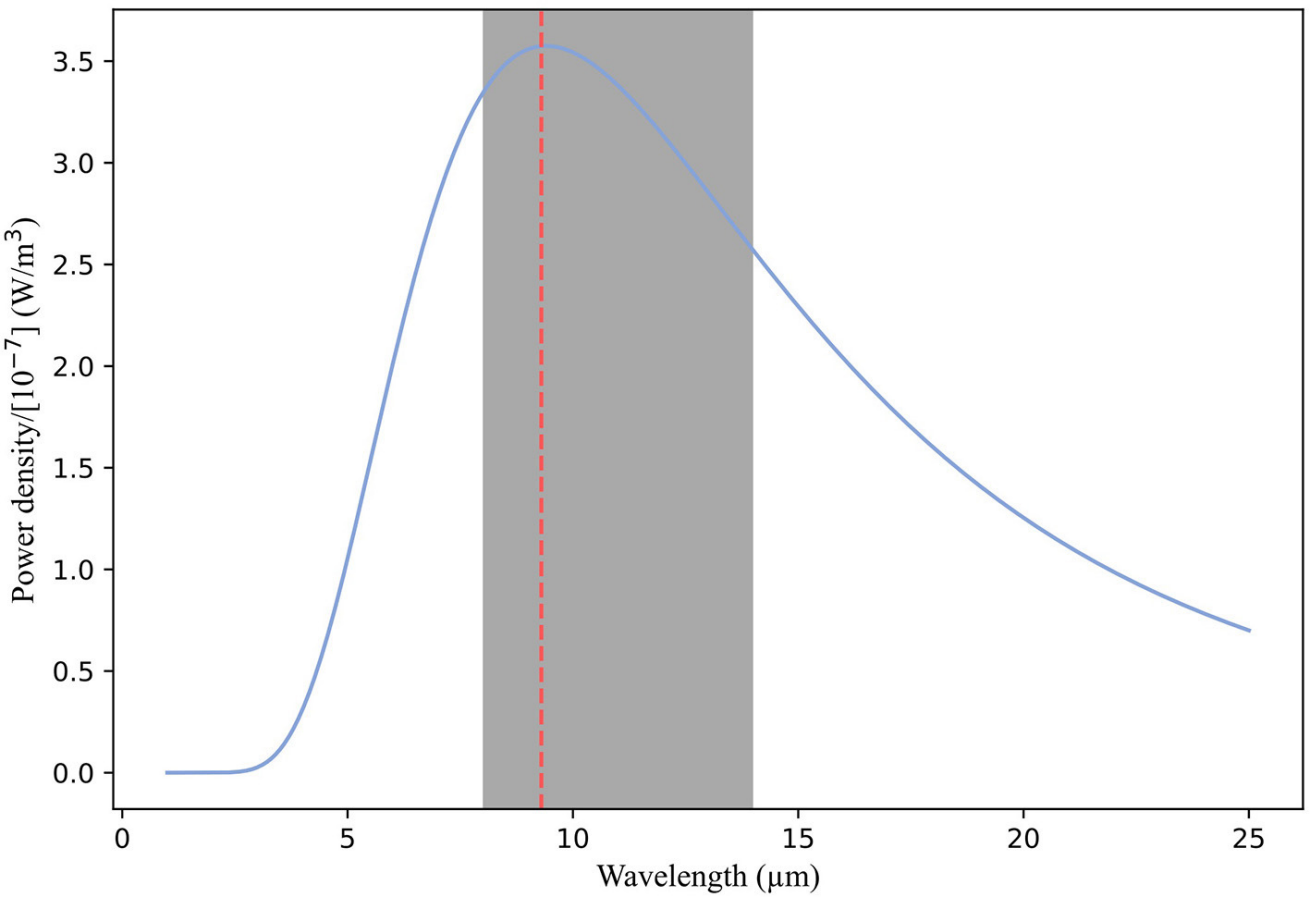
Graph showing the blackbody spectrum for a body at 308.15 K (35°C). For organic tissue at this temperature, thermal energy intensity peaks around 9.3 μm. Cameras with a spectral range including this peak with a wavelength range of 8–14 μm are likely to estimate the apparent temperature well. Cameras covering a more limited wavelength range, such as 3–5 μm, will have to extrapolate to find this peak, leading to greater uncertainty in the final measurement.

Spectral range characterizes the part of the electromagnetic spectrum that a thermal camera can detect. It is the range of wavelengths that the sensor in the camera detects and is measured in micrometers (μm). Apart from gas detection cameras, which operate in the range of 3 to 5 μm, most thermal cameras are longwave, operating between 8 and 14 μm. AAT guidelines recommend a camera detector spectral bandwidth between 8 and 14 μm. Most papers reported the spectral range as 7.5–13 μm (*n* = 13; 11.93%). However, two papers reported spectral ranges outside of AAT guidelines [3–5 μm (114) and 3–5.4 μm (115)]. For organic tissue with a temperature of 35°C (308.15 K), the peak of thermal energy intensity peaks around 9.3 μm (Figure 7). Cameras covering a more limited wavelength range, for example 3–5 μm, will have to extrapolate to find this peak, leading to greater uncertainty in the final measurement.

##### Temperature range

The temperature range is the span of temperatures to which a camera is calibrated and is capable of measuring. Some cameras have multiple ranges to allow for measuring a wider span of temperatures more accurately. However, measuring objects with temperatures at the outer limits of the range leads to loss of image quality. There is an in-camera setting for selecting a narrower range at which the user would like the range to be set. AAT guidelines recommend setting the camera temperature range to cover temperatures within the range of temperatures of the animals to be examined, typically 20 to 45°C. The temperature range was reported in 18 papers (16.36%) and ranged from −40 to 2,000°C. As an example, Lowe et al. (14) utilized a FLIR 650sc thermal camera, employing these limits. However, the datasheet for the camera states that accuracy is ±1°C or 1% of reading for a limited temperature range for objects between 5 and 120°C at an ambient temperature of between 10 and 35°C, and that this is only valid for temperatures within a range of −40 to 120°C. Thus, thermal measurement accuracy in this case is dependent not only on the temperature of the animal but also on the ambient temperature. If the ambient temperature lies outside of the 10–35°C range, thermal measurement accuracy would decrease. Understanding the limitations of the thermal camera being used and ensuring it can measure the temperature in an appropriate range is important to achieve accuracy.

##### Resolution

Resolution determines a camera's ability to generate a high-quality image. The more detector elements (pixels) a thermal camera's detector contains, the higher its resolution and the more detail the image will contain. Pixels are the datapoints for thermal measurement, and these data are used to build an image from the thermal profile. The more datapoints acquired, the higher the resolution and accuracy of the thermal image. Higher resolution IR cameras can detect smaller objects from farther away. Pixel size is responsible for level of detail and is typically measured in μm; a greater number of pixels per unit area will yield superior detail. While resolution was reported accurately in approximately one third of studies (*n* = 39; 35.78%), a few papers used the term ‘resolution' in an alternative sense and reported it as a temperature in degrees Celsius (*n* = 8; 7.34%). Some were incorrectly referring to thermal sensitivity (29, 115) but for the remainder, no corresponding documentation was found to support this error (35, 36, 54, 59, 66, 113). While the AAT recommends an absolute detector resolution of >640 × 480 coupled with a suitable microbolometer and lens, a little over a tenth of papers reported using a camera with this resolution or higher (*n* = 12; 11.01%). High resolution is important, allowing the identification of small thermal details and therefore more accurate temperature measurements, within the same field-of-view.

##### Lens, field-of-view (FOV), instantaneous field-of-view (IFOV), spot size, focal length and focus

The lens influences several parameters including the field-of-view, the instantaneous field-of-view, the spot size and the focus. Lens size (*n* = 8; 7.34%) and field-of-view (*n* = 7; 6.42%) were reported in under 10% of papers (Figure 6). The AAT recommends a lens with a field-of-view of 25°. The field-of-view is the extent of a scene (the imaging area the lens can see) that the camera can see at any given moment. For close-up work, a lens with a wide angle is required (≥45°), while for long distance work a telephoto lens is more appropriate (12° or 6°). Some cameras have multiple lenses to allow for different applications. Reported fields-of-view ranged from 15° to 45° (11, 27, 63, 68, 83, 93, 114). Montanholi et al. reported the use of all available lenses with one of the IR cameras they used in their research (a FLIR SC2000 with 12°, 24° and 45° lenses) and applied each lens at different distances and experimental settings (46). All lenses were used in a ‘distance and IR camera comparison' trial, which demonstrated that temperature varied inversely with distance to subject for all lenses, and that the 24° and 45° lenses showed similar decreases of around 1.5–2.0°C over an increasing distance of 14.5 m (46). The 24° lens was used at distances of 100–150 cm to take images of specific regions of interest such as the snout, the eye and the ear which is an appropriate use of the lens in this context (46). The 45° lens was used for close-up work, 80 cm from the animals, which is appropriate. It was also used from 400 cm away to evaluate the pregnancy status of a heifer (46). While this is not an ideal use of a wide-angle lens, as it was being used for relative temperature measurements to compare flank temperatures, it was appropriate in this context.

A critical parameter that affects how far a thermal camera can see is the focal length of the lens. The focal length of the lens is the distance between the lens and the image sensor when the subject is in focus, usually stated in millimeters. Just one paper reported focal length (0.92%) (64). Focal length determines the instantaneous field-of-view (IFOV or spatial resolution) of a thermal camera system. IFOV is the angular field-of-view of a single pixel and represents the smallest angle that can be resolved by the system, provided there is sufficient thermal contrast. The IFOV can then be used to determine the distance at which a target's critical dimension subtends the required number of pixels to achieve detection, recognition or identification. The longer the focal length of the lens, the smaller the IFOV becomes, which translates into more pixels across a target at a fixed range. The IFOV is measured in milliradians (mRad), and the AAT recommends a spatial resolution quality at eight feet (2.4 m) equivalent to ≤2.6 mRad at 40 cm minimum focus. The few papers which reported IFOV (*n* = 3, 2.75%) adhered to the AAT recommendations (26, 70, 83).

Knowledge and comprehension of spot size, i.e., how much area each pixel covers on the target, is necessary for accurate interpretation of thermal images. The ratio of the maximum distance at which a camera can accurately resolve and measure a minimum spot size to the spot size itself is called the camera's maximum ‘distance to spot size ratio' or simply ‘spot size ratio.' The smallest object of interest must be fully delimited by at least a 10 × 10 pixel grid in order to obtain the closest to absolute temperature accuracy. However, a true measurement (or close to true measurement) may still be possible even if spot size is a single pixel or a 3 × 3 pixel grid. The AAT recommends a minimum measurable spot size of 2.1 × 2.0 mm (3 × 3 or 9 pixels) at 40 cm distance from the animal. Neither spot size nor spot size ratio were reported in any of the articles reviewed. Montanholi et al. mention that temperature likely decreased with increased distance for a particular area due to lowered spot size but did not supply any supporting calculations. In a 2021 thermal study concerning human fever detection and size-of-source effects in IR cameras, Pušnik et al. concluded that in order obtain effective images for disease detection, the operator must account for spot size and that the region of interest must contain at least 10 × 10 pixels of homogeneous temperature of which the area used for temperature should be positioned at least seven pixels from the edge of the region of interest (161).

The camera lens should be focused on the object of interest to yield an accurate measurement. Dunbar et al. found that eye temperatures of well-focused thermograms indicated a statistically similar temperature to core body temperature (63), in agreement with Pušnik et al. (161). Cameras may be fixed focus, meaning they are always in focus, manual focus, meaning the operator must adjust the focus on the camera, or automatic focus, meaning the camera will autofocus based on what it can identify for contrast in the scene. In general, entry level cameras have fixed focus, and high-performance cameras have either manual or automatic focus. Focus was reported in two papers, with one using manual focus (11) and the other using automatic (86). Precision autofocus is preferred by the AAT, which lends itself to automation and frees up the operator to perform other essential tasks and measurements.

##### Camera calibration, stability, drift and accuracy

Calibrating a thermal camera is the process of correlating what the camera sees, in terms of infrared radiation, with known temperatures, so that it can transform the radiation it detects into an accurate temperature reading. All cameras are initially calibrated to factory specifications, but over time, aging of the electronic components can cause calibration shift and produce inaccurate temperature measurements. It is necessary for cameras to be calibrated annually by the manufacturer to maintain their accuracy. Calibration is performed under controlled conditions with multiple blackbodies which are used as reference temperature sources. The theoretical ideal blackbody has an emissivity of 1.0 and perfectly absorbs and emits all radiation. Blackbodies are arranged in a semi-circle and set to different known temperatures, and then the thermal camera, which is connected to a robotic arm, is pointed at each reference source one by one. The signal value at each temperature is captured by calibration software, and each pair of signal and temperature values are plotted along a curve, the equation of which is based on a physics model. This data is then loaded into the camera, calibrating it to ensure it meets accuracy specifications. Because of these extensive requirements, cameras need to be calibrated in a laboratory setting. The AAT guidelines recommend that camera temperature offset should be calibrated against the emissivity of a blackbody at 1.0 if needed for the examination being performed.

While calibration of the thermal camera prior to use was reported in 20 papers (18.35%), on closer inspection it was discovered that the actual method described did not conform to this definition. In most of this subset of papers (*n* = 16; 14.68%) camera calibration was interpreted as either allowing the camera to habituate to the testing environment for 10–15 min, or the operator inputting the recorded relative humidity, ambient temperature and emissivity into the camera prior to use. Of the remaining papers (*n* = 4; 3.67%), one calibrated to a graybody (a body where transmission, absorption, and reflection are all constant with wavelength) prior to use and three were factory calibrated with blackbodies. Ideally, the date of the last laboratory calibration should be reported.

Stability and drift are two main sources of errors in measurement accuracy. Drift was accounted for in two papers (1.83%) and stability was mentioned in one paper (0.92%). Stability is an error that is a function of time, whereas drift is a function of the temperature of the measurement electronics, caused by internal heating of equipment during normal operation or by changes in external ambient temperature. The AAT recommended that thermal drift is strictly controlled by calibration to a known temperature standard if necessary for the study under consideration and advise maintenance of detector uniformity and correction *via* calibration to a known temperature standard, as outlined above. Other 'field' methods include checking the temperature output by the camera against a known temperature, such as that of ice or boiling water. Kammersgaard et al. (55) conducted a stability test post data collection. Using a bucket of icy water as a target, they compared the maximum IR temperature of the water surface (measured using the IR camera) with the surface water temperature (measured using a thermal sensor probe) as a reference temperature. The difference between IR temperature and reference temperature was calculated and it was found on average (over a 90-min period) that the maximum IR temperature was just 0.21°C higher than the reference temperature. The authors concluded that this stability test suggested a superior accuracy of the IR camera to that indicated by the manufacturer. Menzel et al. applied the “difference method” (43, 47), developed by Siewert et al. (162) to account for temperature drift. The method uses the principle that “in the case of two electrical analog signals generated by a sensor and conducted on a printed circuit board which are overlaid with a disturbing signal - caused by electromagnetic coupling into both analog signal lines - through a difference calculation, a strong limitation of the error is achieved” (162). After showing that the difference ROI method could be used for early detection of elevated body temperature in pigs (>39.5°C), Siewert et al. concluded that the difference method is superior to a conventional analysis based on absolute temperature values with error margins in the order of ±2 to ±1.5°C. The method requires that at least two anatomical regions that can be recognized as reproducible are in the IR image, but the authors also state that the results should be interpreted with caution, due to the low sample size of animals used in the study.

Higher-end IR cameras have an internal mechanism to account for drift. A miniature blackbody drops in front of the detector at regular intervals, making a clicking noise when activated. This is called the non-uniformity calibration flag. The camera assumes that this flag has a uniform temperature and corrects for any drift internally. This correction also impacts the temperature measurement and its drift and stability. Ghassemi et al. (163) evaluated the drift and stability of two IR cameras according to the methodology issued by the International Organization for Standardization (ISO:IEC90601-2-59:2017), which outlines the “particular requirements for the basic safety and essential performance of screening thermographs for human febrile temperature screening” (163). They found that neither of the IR cameras satisfied the requirement of both stability and drift being <0.1°C as stated in the international standard without the use of an external temperature reference source within the image (163). This finding is likely to impact the possibility of achieving accurate absolute temperatures in the context of livestock disease detection for all IR cameras.

##### Thermal sensitivity

Thermal sensitivity or noise equivalent temperature difference (NETD) describes the smallest temperature difference that can be captured with a thermal camera. It is a measure of how well a thermal imaging detector can distinguish between very small differences in thermal radiation in the image. The lower the number, the better the thermal sensitivity of the IR system. A temperature-controlled blackbody is used to measure the noise equivalent temperature difference of a detector. The blackbody is stabilized before starting the measurement. The noise equivalent temperature difference is then measured at a specific temperature. It is not a single snapshot measurement, but rather a temporal measurement of noise. Noise is defined as random fluctuation in electrical output from a camera's detector. This noise can interfere with the signal one is aiming to capture. The warmer the detector, the more noise will be present. When the noise is equivalent to the smallest measurable temperature difference, the detector has reached the limit of its ability to resolve a useful thermal signal. The more noise present, the higher the NETD value of the detector. In applications where thermography targets have wide temperature differences, a camera with a low NETD is likely unnecessary. However, for more subtle applications, like different ROIs on a living animal, higher sensitivity would be an advantage. NETD is typically expressed in millikelvin, for example: 60 mK at 30°C, and is sometimes referred to as 'thermal contrast'. While NETD was reported in 24 papers (22.01%), inconsistencies in units were noted, with authors using kelvin, mK or °C. The temperature at which NETD was performed was not recorded in 14 papers and two papers attributed the value to resolution instead of NETD. This reduces the numbers of publications reporting accurate NETD parameters to eight (7.34%). The AAT recommends an NETD of <50 mK at 30°C. Converting all Celsius temperatures provided to mK and assuming all measurements were performed at 30°C, 12 papers satisfied the AAT requirement (11, 14, 15, 17, 19, 22, 33, 36, 52, 83, 86, 98). NETD was correctly recorded as 0.04 K by Hoffman et al. (36) but the temperature at which this was tested (25°C) was not included (36). An earlier paper recorded NETD as 0.08 K but this could not be confirmed on the datasheet; supplied NETDs were 40 mK with the 23° lens, 0.3 K with the 6° lens and 0.1 K with the 41° and 72° lenses (54).

##### Video imagery

The utilization of video vs. still imagery was reported in nearly all publications (*n* = 107; 98.17%) with the latter dominating (*n* = 99; 90.83%). Video recommendations from the AAT are to incorporate real-time image focus and capture capability and that fast frame rate capability is preferred (50 Hz). The higher the refresh rate, the more frames will be produced each second, and the smoother the rendering and therefore the image will more likely be in focus. The refresh rate is measured in Hz, a unit of frequency, denoting the number of times per second one period of an image is cycled. For example, if the refresh rate of a thermal imaging device is 50 Hz, this means that the image refreshes 50 times every second (equivalent to 50 frames/s. Only a minority of studies recorded using video (14, 32, 36, 54, 56, 79, 96, 115) with just one quarter of those using a frame rate >50 Hz (32, 96) due to thermal camera capabilities. The AAT also recommend that for temperature analysis, radiometric video files are used as there is temperature data embedded in each pixel. Use of video rather than still imagery offers the advantage of analyzing more than one image per animal, and shows potential as a monitoring system (14, 54), but there is no research as yet into the optimal number of frames per second for IR video capture in livestock.

##### Camera choice

Camera make and model was reported in 103 papers (94.5%), with 66 of these being manufactured by FLIR. Whether the camera was fixed or handheld was reported in 60 papers (55.05%) with 14 of these being fixed and 46 being handheld. Bleul et al. (9) and Jorquera-Chavez et al. (53) tested a low cost, small, handheld IR camera (FLIR One) attached to a smart phone to assess its capability for disease detection in animals. Both studies found that the camera had limited efficiency. Bleul et al. (9) found it produced large variations in measurements and recommended the use of a higher quality thermal camera for future experiments. Jorquera-Chavez et al. (53) noted in their study that because respiratory rate was analyzed from non-radiometric infrared videos, this could have affected the sensitivity of the measurements due to changes in light conditions.

#### Environmental parameters

**Figure 8:**
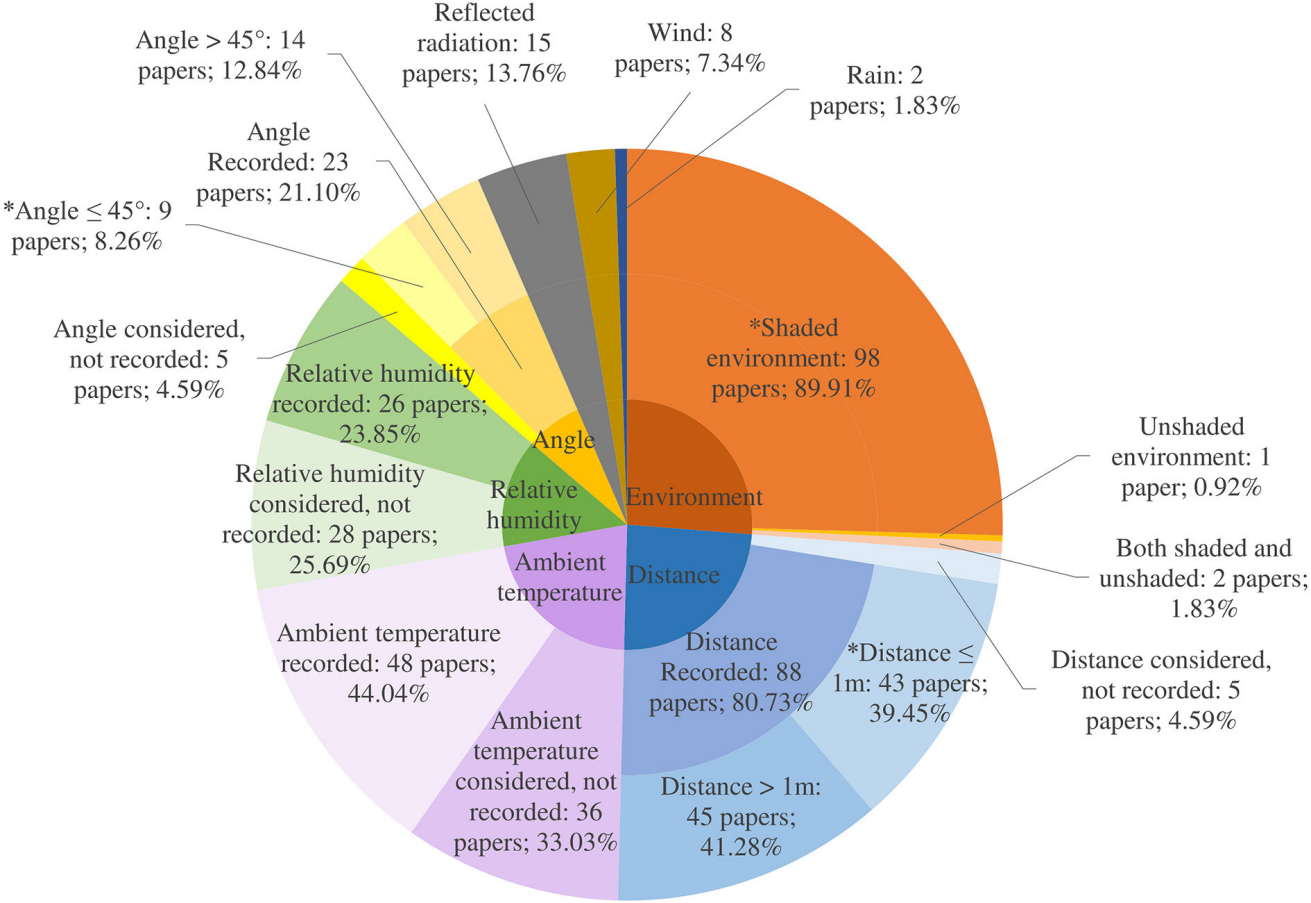
Environmental parameter reporting where ^*^ denotes the recommended value.

Environmental parameters impact IRT temperature measurements significantly and therefore must be managed to ensure reproducible and accurate readings (98). Terrain and climate influence ambient temperature, relative humidity, sunlight, wind and rainfall and IRT is sensitive to environmental changes such as weather fluctuations and radiation from adjacent surfaces. Overall, for the 109 papers in the review, across the 12 environmental parameters, 43.58% of expected data was reported (Figure 8). While some studies incorporated environmental parameters into calculations and analysis, none accounted for all types of environmental parameters, which will be necessary to attain the required disease surveillance accuracy, sensitivity and specificity (31). The camera must be allowed to habituate to the environmental conditions in which it is to be used. To achieve ambient temperature compensation, the Teledyne FLIR user's manual (see text footnote 5). recommends measuring the temperature of the camera and optical path in up to three different locations. These data are then included in the internal calibration equation and can ensure accurate readings through the entire range of operating temperatures (typically −15 to 50°C). This is particularly relevant for using IR cameras outdoors. Additionally, cameras should be given time to warm up prior to taking any measurements. The camera and optics should be kept out of contact with other heat sources or direct sunlight as changing the temperature of either will adversely affect measurement uncertainty.

##### Wind, rain, ambient temperature, reflected radiation and relative humidity

Wind, rain and ambient temperatures can affect animals' skin temperature due to evaporation and blood flow to the skin. Temperature assessments were undertaken in a variety of environments: either indoors in a closed room, outdoors in a shaded barn or in the field in direct sunlight. Church et al. (98) found that in the presence of a 7 km/h wind, mean eye temperature difference was 0.43°C but that at higher wind speeds of 12 km/h, mean difference was 0.78°C when compared with no wind. The presence of wind at both higher and lower speeds resulted in a statistically significant decrease of cattle IR eye temperature. Internal body temperature monitoring revealed that cows exposed to wind and rain could be either hotter or colder than cows indoors due to an increase in the amplitude of the circadian body temperature rhythm (164). Bewley et al. (165) found that both rectal and reticular temperatures were influenced by season, milking, housing system and parity and so IR estimates of these temperatures would be similarly affected. Church et al. (98) worked with different humidity settings on the cameras, finding that as humidity accounts for only a 0.1°C temperature change over its range (0–100% humidity), the setting had very little effect at 1 m at a constant room temperature. As humidity is expected to affect the transmission of IR radiation, humidity corrections may become more pronounced at greater distances and higher temperatures (98). Sunlight and reflected radiation from other surfaces can interfere with the temperature on the IR image collected. For instance, an object that is cool to touch may show a higher temperature on a thermogram if a nearby heat source (such as the operator, or other animals) was reflecting off the surface. Sunlight will also adversely affect the result, with direct sunlight increasing IRT eye temperature by 0.56 ± 0.36°C (98).

##### Distance and angle

Variations in the distance between the subject and the camera as well as the angle of the image can affect the accuracy of the measurements. The AAT recommend a patient to camera distance of between three and eight feet (0.91–2.44 m), as needed to allow the ROI to fill approximately 75% of the image but make no recommendations as to angle of assessment. Church et al. investigated the effect of distance to object and found that as camera to object distance increased from 0.5 to 3.0 m, IR temperature decreased by as much as 2.0°C (98). Okada et al. (166) found that temperatures were significantly lower at distances of 2 and 3 m away from the object of interest (cattle) but that there were no significant differences between distances of 0.5 and 1 m. Just under half of the papers reporting distance used distances of 1 m or less. Playà-Montmany and Tattersall demonstrated that even when incorporating the appropriate object parameters in the thermal image software, increasing the distance away from their calibration source (a blackbody of known temperature and emissivity) led to a linear increase in the apparent error in temperature (167). As the eye to camera distance increased, the IR temperature value decreased for all settings (167).

Ijichi et al. (168) aimed to validate a standardized protocol for the use of IRT eye temperature measurement in mammals with anterolateral eyes, such as horses and cows. In horses, they found a significant strong positive correlation between eye temperature taken from an angle of 90° to the sagittal plane but observed no such correlation from 90° to the nasal plane or eye, suggesting the optimal position would be 90° in relation to the sagittal plane. Jiao et al. found that an angle >45° away from the area of interest yielded significant differences in temperature measurement. Eleven papers reported an angle of 45° or less (33). However, angle reporting was inconsistent: some papers reported a 90° angle to an object, but this was actually a 0° angle to the object and *vice versa*. A paper documenting areas for error when using IRT in ecology found that angles >55° led to a decrease in emissivity when testing with mink fur, along with other biological and non-biological materials (167). Vardasca et al. (169) found that the mean temperature of the inner canthi of the eye of humans varied by 0.1°C at distances between 0.8 and 1.2 m when compared with the reference distance and angle of 1 m and 90°, and by 0.4°C and 0.5°C at angles of 105° and 75°, respectively. They concluded that to minimize measurement error, distances between 0.8 and 1.2 m and angles as close as possible to 90° should be used to obtain the temperature of the medial canthus of the eye. There are now developed methods to account for angles >45° (33). However, where possible, aiming to record thermal images at distances of 0.5–1 m and at angles of 0–45° to the ROI would be ideal. A unified approach to recording angle, with supporting images, would be useful.

## Discussion

### Recommended parameters

In recent years, IRT has progressed as a diagnostic and clinical tool in both the medical and veterinary spheres, although differences exist between these applications. Within a hospital environment, IRT can be performed in highly controlled laboratory-like conditions, with patients capable of describing symptoms to medical practitioners. As such, the use of IRT in humans has become more specialized than in livestock, with the AAT producing separate guidelines for neuro-musculoskeletal^8^, dental-oral and systemic health^9^ and breast health^10^ examinations for cancer screening (170). For use of IRT in livestock, the veterinary AAT guidelines are more geared toward equine thermography, with a focus on sport horses. However, in general terms, indications are similar for use of IRT in humans and animals, i.e., as a diagnostic aid, to enhance clinical assessment and to assess thermal, functional and musculoskeletal stress in animals. While this review focusses on disease detection in livestock, there has also been extensive research undertaken on evaluating IRT to detect heat stress in animals (126, 171, 172) and to assess the reproductive health of sires (173, 174).

Early disease detection using IRT has the potential to yield significant benefits for both animals and humans. A system that uses IRT to detect disease could reduce farm labor, improve animal welfare and increase production yields. To our knowledge, this is the first study to collate and summarize these parameters in an attempt to provide a reference guide. To improve reporting guidelines, researchers should provide parameter data to enable like-for-like comparisons and ensure that the evidence underpinning IRT for use in disease detection for livestock is harmonized, robust, reproducible and repeatable.

Creation of a disease surveillance system incorporating IRT for early detection depends on several parameters to optimize accuracy (see Tables 2–4). These include but are not restricted to: ambient temperature, relative humidity and radiated temperature. In an ideal experimental scenario, the first two can be achieved using a data logger habituated to the environment to record ambient temperature and relative humidity, while the third can be carried out using a Lambert radiator. A Lambert radiator is an object that reflects incident radiation in all directions and represents optimum diffusion. The temperature of the reflected radiation can be measured using a Lambert radiator in conjunction with a thermal camera. Using a crumpled and unfolded piece of aluminum foil (which has a high reflectance and its crumpling allowing for near-perfect diffuse reflection) as a suitable substitute for a Lambert radiator, the foil should be placed on or in the same area as the object one wishes to image. Camera emissivity should be set to 1.0. The temperature of the Lambert radiator as it appears in the image can be input into the thermal camera as the radiated temperature. The camera must be habituated to the environment and ideally personnel trained in IRT should be involved in and/or carry out procedures. Imagery should be collected at 1 m or less and at angles of 45° or less from the ROI.

The IR camera should have a resolution of at least 640 × 480, an NETD of 50 mK at 30°C, a spectral range of 8–14 μm, an IFOV of ≤2.6 mRad at 40 cm minimum focus and a spot size of at least 3 × 3 pixels (ideally 10 × 10 pixels of homogeneous temperature of which the area used for temperature should be positioned at least seven pixels from the edge of the ROI). Blackbody calibration of the camera to reference object temperature and for camera stability and drift should be carried out once per year. Fixed cameras would reduce differences between human operators and would lend the IRT system to automation. The temperature range of the camera and its setting prior to use should include the ranges users are interested in, i.e., between 30 and 45°C, and the lens should have a field-of-view of 25°. If using video, a frame rate of at least 50 Hz and precision autofocus are recommended. However, many commercial thermal cameras offer 30 Hz as the maximum frame rate. For temperature analysis, radiometric files are preferred. However, some video file formats are proprietary and require specific commercial software for analysis. Emissivity values should be established for livestock and researchers should endeavor to obtain training in and understand IR technology prior to its use. Obviously, researchers should strive to achieve *all* ideal parameters when planning future experiments, but practicalities and cost will influence experimental design. Cameras that have the capabilities as outlined above usually cost upwards of $23,000^11^^,^^12^. However, technology is currently advancing at a rapid rate and smaller, lighter and cheaper instruments are being manufactured from year to year and so the ideal equipment is becoming ever more accessible.

### Challenges and opportunities with regards to disease detection

This section discusses the opportunities and challenges that are likely to be encountered in the journey and the design stages of creating an IR system for disease detection. We reflect on three distinct but linked considerations: types of diseases (exotic, endemic and production diseases); absolute vs. relative temperatures; and individual vs. herd measurements, including animal identification, indoor and outdoor environments.

#### Disease types

There is potential for IRT to be used to detect infectious and non-infectious diseases which affect production (mastitis, digital dermatitis), domestic markets [Bovine Viral Diarrhea (BVD), Bovine Respiratory Disease (BRD)] and international markets [notifiable diseases such as FMD and Bluetongue (BTv)]. IRT may be particularly useful for diseases which affect the blood supply or cause focal areas of inflammation such as digital dermatitis, laminitis and FMD of the hood and mastitis of the udder. Nikkhah et al. (67) investigated IRT for detection of inflammation associated with laminitis in dairy cattle, concluding that IRT may have potential as a detection tool for laminitis. Further research was recommended to investigate the relationship between IRT and hoof abnormalities and to identify other diseases affecting hoof temperature.

A 2009 study assessed IRT as a screening tool for FMD, before and after development of clinical signs (107). Eye and hoof temperatures were analyzed and the feasibility of IRT as a screening tool for FMD in cattle was demonstrated finding that IR foot temperatures increased in FMD-infected animals (107). The authors recommended further research to enable differentiation between foot-associated conditions (i.e., to differentiate FMD from digital dermatitis using an IR image) and the development of computational algorithms to assess signature temperature patterns of specific diseases (107). Results demonstrated IRT to be a promising screening technology to quickly identify potentially infected animals for confirmatory diagnostic testing during FMD outbreaks. Dunbar et al. (63) evaluated the use of IRT in detecting heat changes associated with affected sites of FMD infection, namely the feet and mouth, while also recording body temperature from a surgically implanted abdominal temperature logger and eye IRT. In that study, Mule Deer experimentally infected with FMD experienced a significant foot temperature rise over the course of the infection. The authors highlighted that thermograms of the eye may have use in detecting changes in general body temperature by use of the eye as an index of body temperature. IRT may thus have potential as a rapid and remote screening tool for FMD infection and research focused on the correlation of eye temperature and body temperature will determine the practical use of IRT in screening for FMD and other pyrogenic diseases (63). However, a later experiment which evaluated eye and foot IRT in cattle concluded that IRT would “be at best a modest predictive indicator of early FMD” (61). It was noted that activity (or lack thereof) influences the temperature of the hoof and as such a period of acclimatization is required prior to imaging. In agreement with Dunbar et al. (63), this study also suggested that IRT of the eye may be a good proxy for core body temperature (61). The study was completed under ideal field conditions, so this will not necessarily translate to real-life farming and veterinary work (61).

Berry et al. (113) used IRT to gather baseline information on the magnitude and pattern of daily variation in udder temperature and tried to determine the influence of environmental temperature parameters on udder temperature. The authors concluded that IRT showed promise as an early detection method for mastitis when coupled with environmental temperature monitoring. Polat et al. (104) aimed to determine interrelationships among mastitis indicators and evaluate the subclinical mastitis detection ability of infrared thermography (IRT) in comparison with the California Mastitis Test (CMT). The study found that as a non-invasive and rapid tool, IRT could potentially be employed for screening for subclinical mastitis *via* measuring udder surface temperature with a high predictive diagnostic capability, similar to the CMT when microbiological culture is unavailable. Polat et al. stated that the receiver operating characteristic curve “revealed that sensitivity and specificity of IRT (95.6 and 93.6%, respectively) were not different from those for CMT (88.9 and 98.9%, respectively).” In addition to being non-invasive and relatively fast, IRT was sensitive enough to detect thermal changes on udder skin caused by subclinical mastitis (104).

Infrared eye temperature has been highlighted as a potentially useful indicator of core body temperature that is not affected by ambient temperature (61, 63). The temperature of the posterior border of the eyelid and the medial canthus have been found to be the most representative of core body temperature due to rich capillary beds (159). There have been a number of studies investigating eye temperature using IR sensors in cattle (32, 100, 110, 112) to ascertain its feasibility and accuracy for fever, disease, pain and stress detection. Bell et al. (31) found a weak correlation between thermal eye and rectal temperature of 0.28, which is similar to the findings of Scoley et al. of 0.28 (71). Bleul et al. (9), however, established a stronger correlation of 0.37. Bell et al. (31) posit that sex, age and parity of an animal are all differences which warrant consideration in attempting to solve this problem as they likely influence the thermoregulation of an animal. Continuous temperature measurement of individual animals would give confidence that changes in temperature are due to disease rather than natural body rhythms and temperature set points.

Cook et al. (89) investigated radiated surface temperature measurements of groups of piglets and found that a febrile response to vaccination was detectable on thermal imaging when compared with control groups. Two experiments thus far have measured respiratory rate by IRT to assess stress and discomfort in cattle. The first attempted to validate IRT as a method of respiratory rate measurement in adult cows by measuring respiratory rate with continuous IRT imaging of airflow through the nostrils and by counting flank movements from video and live recordings (32). The authors concluded that IRT can be used reliably to measure respiratory rate and that with further development, the technology could be integrated into existing systems for remote monitoring of dairy cows' health and welfare on-farm. The second study recorded air exhaled from nostrils in calves and found IRT to be a suitable method for recording respiratory rate in calves (14). The authors write that once this method is validated, the future application of IRT will rely on the development of algorithms to automate recording and analysis.

#### Absolute and relative temperatures

Farmers and animal health professionals will look to identify normal and abnormal temperatures in livestock, using acquired thermal imagery to differentiate between diseased and non-diseased animals. Fever detection would require absolute temperature detection, meaning a temperature threshold for disease detection would need to be established. However, if one is looking for a change over time, it may be possible to use relative temperatures of individual animals or the herd as a whole. For example, a relative change or increase may be enough to present a signal to detect signs of mastitis or digital dermatitis at an individual animal level, provided animals can be identified. Radiometric JPEGs (RJPGs) are thermal images with temperature information embedded into the image as metadata. These give absolute temperature measurements for each pixel within the image. Systems using RJPGs instead of JPGs will have higher accuracy in terms of temperature measurement but will produce more data for storage and analysis. Systems requiring absolute temperature measurements for livestock should aim to use RJPGs. An option to use relative temperatures instead of absolute would involve building a bank of images over a set time period representing ‘normal' or ‘non-diseased' for an individual animal or for the herd. The IR system would update this bank of images/data once or twice per day and then would be able to identify a change from the norm using relative temperatures. The accuracy of IR cameras must be considered and included in models if disease threshold temperatures are to be established.

#### Individual or herd measurements

Identification of individual animals is another challenge to consider. In the UK, most livestock are identifiable by individual ear tags but only sheep are legally required to have electronic tags that can be read with a radio frequency identification reader. Many farms, however, are already using sensor-based technology for estrus detection, such as pedometers or collars. This ID or the ear tag ID for each animal could be read and uploaded to the system when animals enter the race to identify time of entry and therefore matched with time when the IR image or video was taken. If it is not possible to identify individual animals in this manner, an alternative would be to collect information concerning the whole herd and to monitor for any relative change outside of the norm. As previously mentioned, sunlight and reflected radiation can interfere with the temperature on the IR image. This will likely make outdoor data collection unreliable and further research may conclude that these technologies are only suitable for indoor implementation in, for example, barns and milking parlors. This will have an impact on the species and production type of animal that can be monitored.

### IRT system considerations

Useful real-time reporting would demand on-site (i.e., farm, abattoir) data capture and processing as part of any IRT system. Zhang et al. (175) outlined the necessary components of an IR diagnostic system, including: an IR optical system, an uncooled focal plane array, a signal processing system and a display terminal. Implementation of this system for use with livestock will require some adjustments. Animal identification would ideally be built into the system and the system would need to be autonomous for users to benefit.

Video is considered superior to still imagery as it allows for continuous monitoring of animals as opposed to single time-point measurements, possibly providing more accurate temperature measurements as images are taken multiple times per second, as opposed to single images per animal. IR cameras record at high frame rates, creating large quantities of data, which pose a challenge for data processing, analysis and storage.

Opportunities for data reduction should be explored and exploited. Within the medical field of IRT, researchers apply methods including the subtraction of background from each frame of interest (176), Fast Fourier Transform (117, 177) and Principal Component Analysis (178). Consideration will need to be given to whether the original raw image data will need to be retained in case of future enquiry, for example if there is an exotic disease outbreak and data needs to be kept for future lessons learned or future interrogation.

Color palette is a consideration for processing, illustration and interaction purposes. A ‘false' color scale can be selected to represent the image for thermal image processing. Different color scales are appropriate for different uses. The ‘rainbow' palette is generally the preferred color scale for thermography in medicine and biology (179). Ammer used processed images to demonstrate that false colorings impacted resolution, gray levels and the likeness of the imaged object (179). Howell et al. (180) used the rainbow palette with five cameras from both FLIR and Infratec and found that equine images were incomparable due to different color choices with increasing temperatures from the two manufacturers. They suggested that a solution would be to apply a linear grayscale to all images when thermal images from different software packages are to be compared. Vardasca et al. (181) carried out research into which false ‘rainbow' color scales work best as a visual aid for human interpretation and recommended an internationally accepted standard false color scale to ensure agreement across studies. Olalia et al. (182) found that in hot tropical areas, where the temperature of the scene may match the object being imaged, the more color a palette has, the more noise it generates. As a result, they recommended the use of minimal contrasting colors to render solid detailed images, using palettes such as ‘black-hot' and ‘white-hot'. Ultimately, selection of color palette will depend on the task, the environment and user need. If a machine is interpreting and processing thermal imagery, grayscale may be best, but a ‘rainbow' palette may be more useful to display results to human users.

Cuthbertson et al. (183) explored different avenues to process thermal video imagery of cattle. The authors used line plots to isolate different periods when the animal was either in the field-of-view or not, when the preferred ROI (ocular in this case) was within the field-of-view or whether no animal was present within the field-of-view. Using histograms, they illustrated these three different data scenarios. These mixed distributions allow for fitting of probability density functions to collected video imagery to obtain threshold points so that a data point is assigned to one of three populations. This would enable automation of the system, only collecting the relevant data when the ideal ROI of the animal is within the field-of-view. The authors also had some success with using statistical pre-processing techniques of a 1-s rolling median and quantiles to smooth the temperature data and account for outliers. Outliers in the data were defined as unpredictable movements, such as the animal blinking or shaking its head.

Image analysis techniques have traditionally utilized manual techniques to highlight regions of interest (52) and commercial IR camera software for image processing (100, 110). More recent studies have made advances in terms of advanced statistical predictive modeling (31) and algorithm development (79). To act autonomously and yield real-time results, it is likely that techniques incorporating computer vision and neural networks will be necessary. Jia et al. constructed body temperature prediction models for pigs to compare three machine learning algorithms [Back Propagate Neural Net (BPNN), Random Forest (RF), and Support Vector Regression (SVR)] and found that all three models performed better when environmental parameters were considered and that SVR outperformed the other two models in terms of accuracy (69). Two customized Matlab^®^ algorithms were developed and used by Jorquera-Chavez et al. (53) to process RJPGs and non-radiometric thermal video in cattle. The first algorithm extracted the radiometric information from each image and then selected the ear and eye as ROIs. The second algorithm was used to assess the breathing rate from non-radiometric thermal video, identifying changes in pixel intensity values within the ROI of the nose, which show air exchange during inhalation and exhalation. Computer vision and machine learning techniques are likely to propel this area of research forward in the next few years.

## Conclusion

Precision livestock farming is becoming popular in food production and favors non-invasive techniques of monitoring for animal health and welfare. IRT offers a potential solution using existing technology to monitor and surveil for livestock disease. However, it has been posited that “knowledge of thermal imaging camera accuracy in the scientific literature are limited, poorly investigated and rarely questioned” (161). In this review, we find parameter reporting to date to be insufficient with high numbers of reporting inaccuracies and we recommend that standardized methodology and reporting for IRT measurements are adopted to enable robust comparisons of future investigations. Due to the lack of emissivity values for animals, further research is required to establish accurate values for use with livestock in field settings. It is essential now that research teams attempting to use and validate IR techniques for disease detection include individuals with physics expertise and experience operating IR technology. Without high-level comprehension of the multiple parameters that influence the final image and temperature estimation, it will be impossible to develop reliable disease detection systems. Automation is the obvious next step to achieve functioning IRT systems, but methods need to be tested and validated before this can happen. Managing and processing the large datasets that IRT systems will produce is now possible with sophisticated computer algorithms and machine learning image analysis methods. While research interests range from production to both endemic and exotic diseases with researchers attempting to use both relative and absolute thermal values, IRT may be most useful in its current form to detect mastitis and hoof-related diseases. We conclude that an IRT system to continuously monitor housed livestock as a group or individuals may be the natural next step, with due consideration given to farm layout, animal identification and specific diseases that are important to detect.

## Author contributions

RM was responsible for the conception, data collection, data analysis, and composition of the paper. All authors contributed to manuscript revision, are accountable for the accuracy and integrity of this work, and have approved the final manuscript for publication.

## Funding

This study was supported by a University of Glasgow Vet Fund PhD studentship.

## Conflict of interest

The authors declare that the research was conducted in the absence of any commercial or financial relationships that could be construed as a potential conflict of interest.

## Publisher's note

All claims expressed in this article are solely those of the authors and do not necessarily represent those of their affiliated organizations, or those of the publisher, the editors and the reviewers. Any product that may be evaluated in this article, or claim that may be made by its manufacturer, is not guaranteed or endorsed by the publisher.

## Footnotes

^1^American Academy of Thermology. Guidelines for Veterinary Thermography (2022). https://aathermology.org/wp-content/uploads/2018/04/Guidelines-for-Veterinary-Thermography-2022.pdf [accessed June 6, 2022].

^2^Teledyne FLIR LLC. FLIR Txxx Series User Manual. https://support.flir.com/DocDownload/Assets/dl/1558792.pdf [accessed June 6, 2022].

^3^Teledyne FLIR LLC. FLIR ix User Manual. https://support.flir.com/DocDownload/Assets/dl/t559733-en-us_a4.pdf [accessed June 6, 2022].

^4^Infrared Integrated Systems Ltd. (Irisys). IRI 4010 User Manual. https://www.manualslib.com/manual/1012541/Irisys-Iri-4010.html [accessed June 6, 2022].

^5^Teledyne FLIR LLC. FLIR Ex Series User Manual. https://www.flir.com/globalassets/imported-assets/document/flir-ex-series-user-manual.pdf [accessed June 6, 2022 ].

^6^Teledyne FLIR LLC. FLIR ix User Manual. https://support.flir.com/DocDownload/Assets/dl/t559733-en-us_a4.pdf [accessed June 6, 2022].

^7^IRISYS. IRI 4010 User Manual. https://www.manualslib.com/manual/1012541/Irisys-Iri-4010.html [accessed June 6, 2022].

^8^American Academy of Thermology. Guidelines for Neuro-Musculoskeletal Infrared Medical Thermology (2022). https://aathermology.org/wp-content/uploads/2018/04/AAT-Oral-Systemic-Guidelines-2022.pdf [accessed July 6, 2022].

^9^American Academy of Thermology. Guidelines For Dental-Oral And Systemic Health Infrared Thermography (2022). https://aathermology.org/wp-content/uploads/2018/04/AAT-Breast-Guidelines-2021v2.pdf [accessed July 6, 2022].

^10^American Academy of Thermology. Guidelines for Breast Thermology (2022). https://aathermology.org/wp-content/uploads/2018/04/AAT-Breast-Guidelines-2021v2.pdf [accessed July 6, 2022].

^11^Teledyne FLIR LLC. FLIR A615. https://www.flir.co.uk/products/a615/?vertical=rd%20science&segment=solutions%20 [accessed June 8, 2022].

^12^PASS Equipment and Accessories. Price of FLIR A615. https://www.tester.co.uk/flir-a615-automated-process-r-d-thermal-camera-w-choice-of-lens [accessed June 8, 2022].
