# Supplementary material for: Thermography for disease detection in livestock: A scoping review
Source: Front Vet Sci. 2022 Aug 9;9:965622. doi: 10.3389/fvets.2022.965622 (PMC9395652; doi:10.3389/fvets.2022.965622)
Supplement: Supplementary file 1 [file Data_Sheet_1.docx]

Supplementary Material

# Supplementary Data

Supporting calculations for Table 5

Emissivity formula: $E=\epsilon\sigma T^{4}$

Where,

E = energy flux (also called the radiant emittance),
ε = emissivity, 
$\sigma$ = Stefan–Boltzmann constant (5.67x10-8 W m^-2 K^-4) and
T = Temperature (in kelvin).

or equivalently: $T=\left( \frac{E}{\epsilon\sigma} \right)^{\frac{1}{4}}.$

Error propagation formula: $\delta f=\left[ \sum_{i} \left( \frac{\partial f}{\partial x_{i}}\delta x_{i} \right)^{2} \right]^{\frac{1}{2}},$

applied to the temperature-emissivity equation:

$$\delta T= \sqrt{\left( \frac{\partial T}{\partial\epsilon} \delta\epsilon\right)^{2}+\left( \frac{\partial T}{\partial E} \delta E \right)^{2},}$$

where:

$$\frac{\partial T}{\partial\epsilon}= -\frac{\epsilon^{-\frac{5}{4}}}{4}\times\left( \frac{E}{\sigma} \right)^{\frac{1}{4}}, and \frac{\partial T}{\partial E}=\frac{E^{-\frac{3}{4}}}{4\epsilon\sigma} .$$

Assuming $\delta E=0$, i.e. ignoring the uncertaintity in the measured emission, we have:

$$\delta T= -\frac{\epsilon^{-\frac{5}{4}}}{4}\times\left( \frac{E}{\sigma} \right)^{\frac{1}{4}}.$$

For a given temperature, 308.15K (35^o^C), and considering the Stefan Boltzman constant $\sigma=5.67 {10}^{-8}W/m^{2}K^{4}$, we can obtain the error in temperature for different choices of $\epsilon$. See Table 5.
